# Mumps Virus: Replication, Immune Response, and the Changing Landscape of Vaccine Effectiveness

**DOI:** 10.3390/pathogens15010072

**Published:** 2026-01-09

**Authors:** Jacquline Risalvato

**Affiliations:** Biomedical and Diagnostic Sciences, College of Veterinary Medicine, The University of Tennessee, Knoxville, TN 37996, USA; jrisalva@utk.edu

**Keywords:** mumps virus (MuV), Paramyxoviridae, RNA virus replication, host–virus interactions, viral innate immune responses, viral adaptive immune responses, vaccine mismatch, vaccine efficacy

## Abstract

Mumps virus (MuV) is a single-stranded, negative-sense RNA virus of the Family *Paramyxoviridae*. MuV is a highly contagious human pathogen that causes primarily mild symptoms, including hallmark swelling of the parotid glands. Severe cases can occur, leading to neurological complications, including deafness, meningitis, and encephalitis. The mumps vaccine, now included in combination with measles and rubella vaccines (MMR), was first made available in the 1960s. After its introduction, mumps incidence dropped dramatically to less than 500 cases annually in the US. However, even with long-standing vaccination programs, MuV continues to challenge the landscape of public health due to a resurgence of cases in the past several decades and a still present lack of approved antiviral drugs and treatments available for the disease. This review will explore the biology of MuV, focusing on how MuV replicates and interacts with the host immune system. Recent studies have also shed light on the role of protein phosphorylation in regulating viral RNA synthesis—particularly the dynamic interactions between the nucleoprotein (NP) and phosphoprotein (P)—offering new insights into how the virus controls its replication machinery both mechanistically and through utilizing host cell advantages. We also examine how the immune system responds to mumps infection and vaccination, and how those responses may vary across viral genotypes. Although the Jeryl Lynn vaccine strain has played a key role in controlling mumps for decades, outbreaks among vaccinated individuals have raised questions about the present vaccine’s efficacy against circulating and emerging genotypes and if novel strategies will be required to prevent future outbreaks. We review current epidemiological data, highlighting shifts in MuV transmission and genotype distribution, and discuss the need for updated or genotype-matched vaccines. By connecting molecular virology with real-world trends in disease spread and vaccine performance, this review aims to support ongoing efforts to strengthen mumps control strategies and inform the development of next-generation vaccines.

## 1. Introduction

A member of the viral Family *Paramyxoviridae*, subfamily *Rubulavirinae*, and genus *Orthorubalavirus*—Mumps Virus (MuV) [1,2] is primarily a human pathogen possessing a nonsegmented, negative-sense, single-stranded RNA genome of 15,384 nucleotides. Other relatives of MuV include Newcastle Disease Virus of poultry, Sendai virus of mice, and Measles virus of humans [3]. MuV is most closely related to Parainfluenza virus 5 (PIV5), with over 60% amino acid genetic relation [1,2]. Mumps disease is a febrile illness often characterized by the infamous swelling of the parotid glands, but more severe may result in meningitis, encephalitis, and deafness. It has also been documented that one-in-four post-pubescent males when infected with the virus will develop orchitis, with over 10% of these patients developing resulting subfertility [4,5]. The disease is primarily transmitted through the upper respiratory tract or contact with the conjunctivae by droplet transmission [6,7].

First described by Hippocrates in the book Epidemics in 5th century BCE, the Mumps disease was characterized by parotitis and complicated by orchitis in both children and adults [7]—suggesting that the ailment entered the human population as early as 5000 years ago [8]. In 1790, the physician Hamilton first documented the disease inciting central nervous system (CNS) involvement [9,10], and by this time the disease was given the name “mumps” which is thought to have meant “to sulk” or “to talk indistinctively” in the old English or Scottish language—likely representative of the increased swelling of the parotid glands impairing the ability of patients to speak properly [11,12]. In World War I, mumps rose through the ranks as one of the top diseases causing hospitalization among soldiers—leading to many outbreaks given the proximity of the soldiers in the trenches and deployment environment [7].

Roughly 150 years after gaining its name, the etiological agent of Mumps was discovered. Researchers Claud Johnson and Ernest Goodpasture in 1934 confirmed that a virus was responsible for mumps via the demonstration of the disease in rhesus macaques after injection with saliva from diagnosed human mumps patients. Through the transference of saliva from these infected rhesus macaques to children and back to naive macaques under bacteria-free conditions, the occurrence of disease would be confirmatory for viral etiological agent identification [13]. It would not be until 1945, though, that Mumps virus (MuV) would be isolated and propagated in embryonated chicken eggs—which represented a major step in biological researchers to be able to characterize and study the virus in culture [14,15]. This progress exhibited an additional milestone in MuV’s characterization, as it was discovered that serial passaging MuV in eggs lead to viral attenuation—and vaccine development.

The first MuV vaccine was developed in 1967 using the strain “Jeryl-Lynn”, named after Jeryl Lynn Hilleman, daughter of microbiologist and vaccinologist Maurice Hilleman who was a researcher for Merck at the time. Hilleman isolated the agent from his daughter’s throat during a bout of mumps and used that sample to isolate and culture an attenuated strain for the MuV vaccine we still use today [16]. After successful licensure in 1971, implementation of a two-dose vaccine series in the US in 1989 lead to over a 99% reduction in the number of cases reported annually with less than 0.1 mumps cases per 100,000 people by 2001 [7,17,18]. However, since 2006, notable outbreaks have reemerged among highly vaccinated young adult populations [17]. These events are attributed in part to waning immunity several years after childhood vaccination and to antigenic differences between the vaccine genotype A strain and currently circulating genotypes (such as G and H), which may modestly reduce neutralization efficiency. Together, these factors underscore the need for enhanced viral characterization and updated approaches to MuV outbreak control.

In this review, we examine the mumps virus (MuV) from the perspectives of molecular biology, immunology, and public health. We begin by discussing the mechanisms of MuV replication, including the interactions between viral nucleoprotein and phosphoprotein as well as new discoveries regarding protein phosphorylation and replication. Next, we will investigate how the host immune system reacts to vaccination and infection, and how these reactions can vary depending on viral genotype. Lastly, we will evaluate the state of MuV outbreaks, especially in vaccinated populations, and current epidemiological trends and their implications on human population health and current vaccination dynamics. In conclusion, we will assess the efficacy of the established Jeryl Lynn vaccine considering new genotypes and discuss the difficulties and possibilities for future vaccine development. When combined, these viewpoints seek to offer a thorough, comprehensive investigation into MuV as an impactful virus of human disease and educate in efforts to strength mumps control strategies in an ever-changing landscape.

## 2. Mumps Virus Genome Organization and Structure

### 2.1. MuV Structure and Morphology

**Figure 1:**
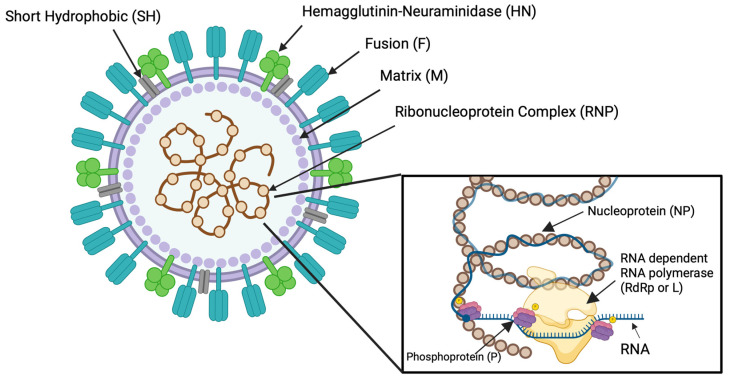
Visual representation of the mumps virus virion including the two glycoproteins studding the virus surface—hemagglutinin-neuraminidase (green) and fusion proteins (aqua). The matrix protein (purple) creates the shell of the virion and is embedded with short hydrophobic protein (gray) throughout. The nucleoprotein (brown) forms a helical capsid structure. Zoomed in, this structure of the nucleoprotein and RNA genome (blue) are visualized with a RNA dependent RNA polymerase (yellow) attaching itself to the RNA genome for replication and chaperoned by the tetrameric phosphoprotein (pink and purple), which is phosphorylated (yellow circle with interior letter P attached to tetramer).

The MuV virion was successfully captured by electron microscopy in the 1960s, where it was found to be enveloped, pleomorphic, and ranged anywhere from 100 to 600 nm in size [19,20]. The matrix (M) protein of MuV is what forms the structured virion shell underneath the host cell-derived lipid envelope. Embedded within the viral membrane is the small hydrophobic (SH) protein, which plays a role in viral exocytosis [21]. The virus particle’s “studded” appearance is due to the two outer glycoproteins, hemagglutinin-neuraminidase (HN) and fusion (F) proteins. These outer glycoproteins project up to 15 nm from the enveloped viral surface [19]. On the inside of the virion lies the ribonucleoprotein (RNP) complex, formed by the nucleoprotein (NP) and the viral RNA genome. NP encaspidates the viral RNA genome by forming a left-handed, helical nucleocapsid 0.98 µm in length [22]. One nucleocapsid ring is formed by thirteen NP subunits, along with 78 nucleotides of the viral genome—a design by which upholds the paramyxovirus “Rule of Six”. The “Rule of Six” is defined by the number of nucleotides within the viral genome being a multiple of six to maintain full encapsidation of the RNA genome by each NP subunit, as each subunit binds to exactly six nucleotides. This “Rule of Six” is a structural feature of all paramyxovirus genomes and their nucleoprotein capsids [23,24,25]. These nucleocapsid rings stack upon one another to form a long helical capsid with a total diameter that ranges from 17 to 20 nm [26]. Figure 1 showcases a visual representation of the MuV virion and the RNP complex with its RNA-dependent RNA-polymerase (RdRp).

### 2.2. Mumps Virus Genome Organization

**Figure 2:**
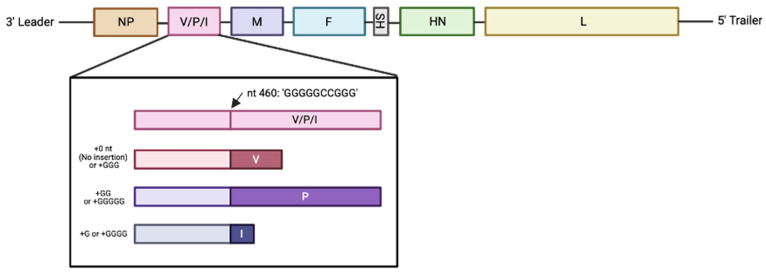
A “block” representation of the mumps virus genome including the 3′ Leader, NP-V/P/I-M-F-SH-HN-L, and 5′ Trailer. Also shown is the guanine nucleotide rich region of the V/P/I transcriptional unit and the guanine nucleotide insertions required for the frameshift of V into either the P or I transcripts.

**Figure 3:**
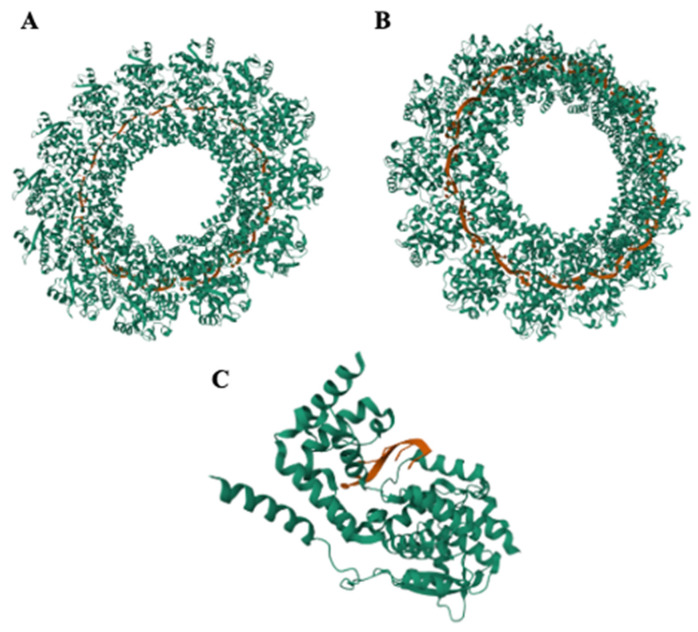
The cryo-EM image of the mumps virus nucleoprotein in both its ring form and individual states from Shan et al., 2021 [34]. Protein is shown in teal, and RNA is represented in orange. (**A**,**B**) are the top-down and underside views of the helical ring of mumps nucleocapsid, respectively, based on the Worldwide Protein Databank (PDB) structure 7EWQ [36]. (**C**) is an individual mumps virus nucleoprotein, where the RNA rests on a divot within the nucleoprotein structure and covered by what is referred to as a “hinge” region thought to mobilize for the needs of the viral RNA-dependent RNA-polymerase during genome replication. This structure is based on the PDBB structure 7EXA [37].

The MuV RNA genome is nonsegmented, single-stranded, and negative-sense. At 15,384 nucleotides in length, there are seven transcriptional units identified: a 3′ Leader sequence followed by NP, V/P/I, M, F, SH, HN, L, and flanked by a 5′ Trailer sequence (Figure 2). The seven transcriptional units of the MuV genome encode for nine proteins, three of which are transcribed by the V/P/I gene via RNA editing [3,27]. The V protein’s transcript is the “true” transcript of this transcriptional unit, while the insertion of either two or four non-template guanine (G) residues into a G-rich coding region in the cysteine-rich open reading frame of V results in the formation of the P and I mRNA, respectively [28]. The transcription of P is due to a +2-reading frame shift, while the transcription of I is due to a +1 or +4-reading frame shift (Figure 3). V, P, and I all possess the same amino (N)-terminus (or NTD), but different carboxy (C)-terminus (or CTD) regions due to this frame shift. The use of the V/P/I open reading frame (ORF) in MuV exemplifies how viral genomes can encode multiple proteins from a single genomic region, thereby maximizing coding efficiency without increasing genome size.

The leader sequence of MuV is 55 nucleotides long, while the trailer sequence terminates the genome with 24 nucleotides. The transcription and replication of this virus are governed by the 3′ to 5′ orientation of its transcriptional units, resulting in higher expression levels of genes located near the 3′ end of the genome compared to those positioned downstream. The gene start and end sequences flanking each transcriptional unit signal the viral RNA-dependent RNA polymerase (vRdRp or L protein) to initiate mRNA synthesis, polyadenylation, and termination. The non-coding regions between these units, known as intergenic regions, consist of nucleotide sequences that are not transcribed into mRNA and thus do not contribute to protein synthesis.

#### 2.2.1. Nucleocapsid Protein (NP)

The nucleocapsid protein (nucleoprotein or NP) is the most abundant protein of MuV and the first transcribed gene within the MuV genome. At 549 amino acids in length and 61 kD in size, there are two main domains of NP: The N-terminus (head) and C-terminus (tail) [29]. Another denotation of these regions is NP_NTD_ and NP_CTD_, respectively. NP_NTD_ is the largest portion of the NP at 400 amino acids in length and is often referred to as the “assembly domain” due to its role in the encapsidation of the RNA genome and the formation of the nucleocapsid. Also, the N-terminus is responsible for the binding of the phosphoprotein (P), which will be discussed later. The interaction between NP_NTD_ and P is what allows the viral polymerase to associate with NP and thus recognize the RNA genome for replication [30,31,32]. Likely due to its essential roles in viral genome replication, the NP_NTD_ is conserved; however, NP_CTD_ is reportedly hypervariable and considered to be intrinsically disordered in its structure [19,26,33,34]. Furthermore, the work of Li in 2009 [35] showed that not only are MuV NP, M, HN, and F required for virus-like particle (VLP) formation, but that the last 15–30 amino acids of MuV NP and its interactions with M are required for trafficking and construction of VLP formation. Additionally, this appears to be a transient property that can be utilized interchangeably with other paramyxovirus proteins as well, such as PIV5 [35].

The ring structure of MuV’s NP to encapsidate the nucleocapsid is essential for its scaffolding actions to enhance RNA replication and transcription. A conformation change in the MuV NP is required to switch between different functions, which is initiated by the NP_CTD_ as a regulator of helical nucleocapsid formation between the dense and hyperdense states of the helical pitches. The NP has structurally plasticity and is unique in its ability to switch between forms to fulfill the roles of either transcription or replication of the genome and its condensation needs [34]. Figure 3 shows the published cryo-EM structure of the nucleocapsid ring and an individual NP holding onto an RNA template.

When it comes to negative-stranded RNA viruses, NP’s primary function is to encapsidate the viral genomic RNA via the formation of the nucleocapsid. During viral mRNA synthesis and genome replication, the NP binds to both the viral genome and antigenome regardless of sequence specificity—a hallmark of negative-stranded RNA virus NPs [38]. Due to the NP structure’s nuclease resistance features, the RNA genome is protected from innate immune defenses by the host cell when encapsidated. During replication, the helical nucleocapsid must be uncoiled by the N-terminus of MuV’s P (or P_NTD_) via a NP-P interaction that allows for the viral RdRp (vRdRp) to access the viral genomic RNA template [26,39]. It is also known that the phosphorylation of NP has been shown to play a role in the synthesis of viral RNA and the genome’s stability [40,41].

If existing without bound RNA, the nascent NP (NP^0^) exists in a soluble form when within the cell. During viral transcription, the phosphoprotein binds to a NP monomer to form a NP^0^-P complex where the P acts in a chaperone-like capacity to prevent NP^0^ from accidental encapsidation of cellular RNA in the cytosol [42,43]. When a sufficient number of NP^0^-P complexes have accumulated within the cytosol, a critical mass point is reached that triggers a “switch” from viral transcription to replication and initiates the formation of a new nucleocapsid by the NP^0^ molecules to encapsidate the now abundantly available MuV RNA genome [44,45].

#### 2.2.2. Phosphoprotein (P)

The phosphoprotein (P protein) of MuV is made through a frameshift in the V/P/I transcriptional unit by an insertion of two non-template guanine (G) nucleotide residues in a G-rich region (Figure 2). Once translated, P is 391 amino acids in length and roughly 47 kD in size [46]. P acts as a cofactor and chaperone of L protein and NP functions and has numerous critical roles in both virus replication and transcription [47].

P, as its name suggests, is a highly phosphorylated protein that undergoes phosphorylation by host cell kinases at its serine and threonine amino acid residues. It is thought that P phosphorylation is essential for the regulation of certain aspects of MuV RNA synthesis [47]. Polo-like kinase 1 (PLK) and ribosomal protein S6 kinase beta-1), for example, are two human host cell kinases known to phosphorylate MuV P and negatively regulate MuV transcription [48,49], displaying P’s role as a key regulator of MuV transcription and replication.

While P itself does not have intrinsic enzymatic activities, its interactions with NP and L are adaptive for the RNP complex. Acting as a chaperone, P interacts with L polymerase to assist with its access to the NP and RNA template to allow for genome transcription and replication [50]. Further, P binds to the NP within both the NP_NTD_ and NP_CTD_s [39,51]—a feature unique to MuV P that has not been documented in other paramyxoviruses. NP and P binding interactions are essential for successful viral replication and transcription of MuV. As previously mentioned, the nascent NP self-assembly and accidental encapsidation of host cell RNA is also prevented by P binding to NP^0^ [42,52]—so P not only assists in viral transcription and translation progression, but also in preventing in accidental inhibition of the process through nondescript RNA binding. Importantly, the ability of P to maintain NP in this RNA-free NP^0^ state depends on the multivalent nature of the P tetramer: its oligomeric arrangement positions multiple NP-binding domains in the proper spatial orientation, allowing P to engage NP across several surfaces with high avidity and thereby block premature NP–NP assembly and nonspecific RNA interaction. Additionally, P binding to NP can provide an “anchoring” mechanism for the vRdRp to the NP^0^RNA template, which assists in less replication errors in MuV genomic copies and adherence [39,50].

**Figure 4:**
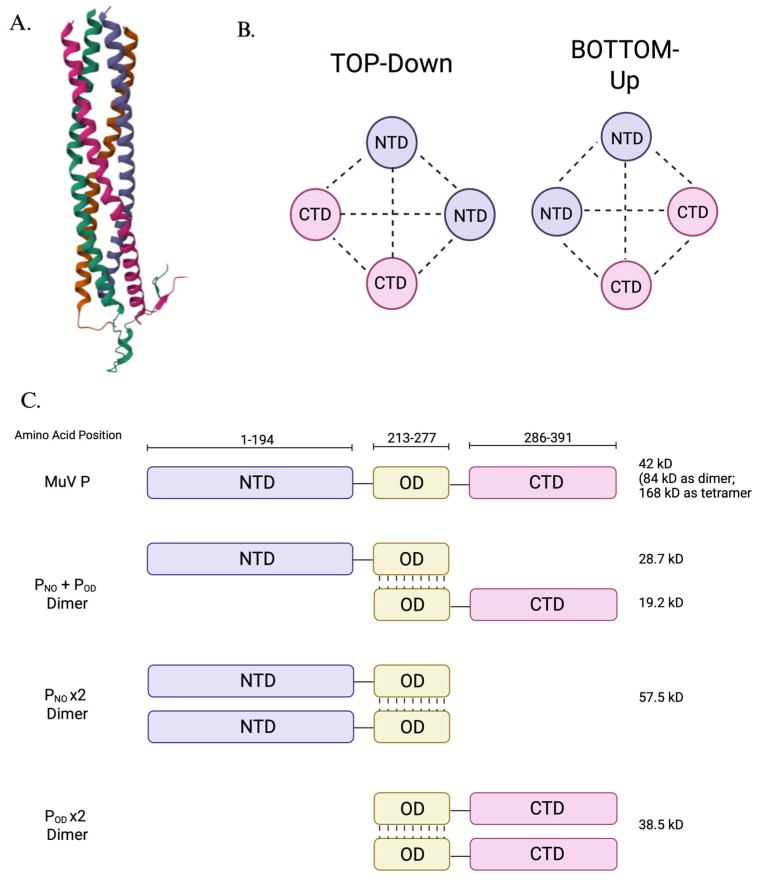
Mumps virus phosphoprotein (P) as a tetramer and its dimeric interactions. (**A**) is the cryo-EM image from Li et al., 2024 [53], showing amino acids 214–304 (the oligomerization domain and part of the carboxy terminal domain of P). There are 4 chains shown in orange, green, pink, and purple. This structure is based on the PDBB structure 8XYO [54]. (**B**) is a top down (**left**) and bottom up (**right**) representation of how the amino (NTD) and carboxy (CTD) terminal domains interact with one another. P’s tetrameric formation is based on a pair of monomers forming a dimer, and those dimers interacting with one another in anti-parallel. However, the top and bottom formation of where the NTD and CTD lie in the formation of the tetramer vary due to oligomeric domain interactions. (**C**) is a visual representation of the dimeric formations based on the oligomerization domain. In Pickar et al., 2015, it was found that the P_NO_ and P_OC_ could interact in parallel or antiparallel dimers with one another [51]. Created in BioRender. Risalvato, J. (2025) https://BioRender.com/61gzo48.

P’s structure is truly unique among paramyxoviruses as it is a tetramer formed by a pair of P dimers oriented in anti-parallel to one another—or two pairs of parallel dimers in anti-parallel (Figure 4)—and is thought to be essential to its stability and role in NP and L interactions during replication. Pickar et al. (2015) showed that when P is split into only its N-terminal and oligomerization domains (P_NTD-OD_) and the oligomerization domain and C-terminus (P_OD-CTD_), these modified proteins interact with the oligomerization of one another’s P constructs and can act independently or cooperatively to enhance MuV genome replication. This tetrameric coiled-coil (or, two homodimers in anti-parallel forming a tetrameric structure) architecture is also crucial for regulating NP’s RNA-binding competence: by organizing its interaction domains in a rigid, multivalent arrangement, the P tetramer can both stabilize NP^0^ and, upon engagement of the polymerase complex, undergo conformational changes that facilitate the controlled transfer of NP onto the nascent viral RNA. In this way, tetramerization is directly tied to P’s ability to govern when and how NP becomes RNA-bound during nucleocapsid assembly [51].

#### 2.2.3. V Protein

The V protein is 25 kD in size and 224 amino acids in length. V is a non-structural protein produced during the infection phase of the virus life cycle and “faithful” transcription by the V/P/I transcriptional unit (736). Through the cytosine-rich region of V (which is also responsible for the vRdRp stuttering and accidental insertion of additional G residues resulting in either P or I transcripts), V binds to melanoma differentiation-associated protein 5 (MDA5) and blocks both interferon (IFN) expression and interleukin-6 (IL-6) signaling by the host cell via degradation of STAT-1 and-3 [55,56,57,58].

MuV V is known to be incorporated within MuV virions in a fashion similar to another paramyxovirus, PIV5. Incorporation of V is believed to allow for MuV replication once inside the host cell by blocking the innate immune pathways and delaying degradation of virion proteins needed for the replication process. As such, V plays a critical role in MuV pathogenesis [59]. This incorporation of V into the virion is hypothesized to be done via NP interactions since the P_NTD_ (shared with V) is known to interact with NP. V protein has also been shown to interact with the MuV replication complex, directly involving itself with the transcription and replication process of MuV [60]. It has also been shown by Xu et al., 2012 that deletion of the V protein of MuV attenuates the virus in tissue culture, speaking to V’s role in MuV virulence [59].

#### 2.2.4. I Protein

I protein at 19 kD and 171 amino acids in length is the smallest of the three proteins derived from the V/P/I transcriptional unit and arises from a shift in the V/P gene ORF via addition of one G residue at the G-rich editing site. The I protein of MuV (or C, or W protein as it has also been named in the literature) has an indeterminate role—but I polypeptides have been detected in MuV-infected cells [28,61]. Though I is not required for virus replication, its shared N-terminus with V and P alludes to its possible involvement in either immune-mediate responses (via interactions with V) or replication (via interactions with P and NP) [62].

#### 2.2.5. Matrix Protein (M)

Mumps virus matrix (M) protein acts as the shell of the virion and is essential for the process of both viral budding and assembly of virions in the host cell. M is 375 amino acids in length and 40 kD in size [46,63]. M forms the inner layer that lies beneath the external lipid membrane acquired from the host cell during viral egress. M protein folding creates an external capsid surface of positively charged domains that allows for binding to the negatively charged lipid envelope [64]. By interacting with NP and the fusion (F) glycoprotein, M protein allows for MuV particle budding out of the host cell. Additionally, M’s interactions with NP and P are required for assembly as M recruits and interacts with host cell proteins to assist in gathering viral components at the plasma membrane surface to increase protein proximity to assemble virus particles and bud from the cell [65,66,67]. M additionally contains late domains, which have a role in the later stages of viral budding—amino acids FPVI and FPIV motifs in MuV and PIV5 M, respectively, and are essential for M’s interaction with host proteins at the plasma membrane surface [65,68].

#### 2.2.6. Fusion Protein (F)

MuV fusion (F) protein is a glycoprotein that is also a type-I transmembrane protein that facilitates virus-to-cell and cell-to-cell membrane fusion. In its inactive precursor form, F_0_, it is 538 amino acids in length and just over 59 kDa in size [69]. F_0_ undergoes N-glycosylation in the rough endoplasmic reticulum and is then cleaved by host endoprotease furin within the Golgi apparatus at amino acid residues 98–102, specifically the R-X-L/R-R motif (e.g., RRHKR) [70]. This cleavage yields the F_1_ and F_2_ subunits, which are linked by disulfide bonds. The F_1_ subunit is approximately 48 kDa, while the F_2_ subunit is smaller at 9 kDa [71,72]. The role of F is to mediate the fusion of the lipid membranes of host cells, which occurs at a neutral pH. Binding of the second glycoprotein on MuV’s surface, hemagglutinin-neuraminidase, to a receptor on the host cell surface signals the F protein to undergo a conformational change required for driving fusion of the virus envelope and cell membrane [73,74].

The first nineteen amino acid residues of the MuV F are a signal peptide that promotes F protein function [63,75] and varies between strains, compared to F’s conserved AA 98–102 domain [76]. The “anchor” point of MuV F lies in amino acid residues 483–512 and tethers F to the viral matrix. There is very little variation between MuV strains and this F anchor domain [63]. While amino acid 195 is known to play a role in MuV fusogenicity, it is not strongly conserved amongst strains—leading to virologists to hypothesize if this residue change from “older” Jeryl-Lynn era strains to what is observed in the more recent strains assists in any capacity with neutralizing immune responses [76]

This cleavage and heterodimer formation is required for F’s “fusogenic” activity through the exposure of a hydrophobic domain within the amino terminus of F_1_. This hydrophobic region, composed primarily of hydrophobic amino acid residues, is found to be relatively conserved amongst paramyxoviruses. Within the F1 ectodomain of MuV, there are heptad repeats of these hydrophobic residues—which is a repeat of a sequence of seven amino acid residues in various patterns that assist in forming stabilized helical bundles in viral proteins. Heptad repeats 1 and 2 (HR1 and 2) form a stabilized six-helix bundle within the F glycoprotein that is directly involved with MuV fusion. The precise mechanisms of this HR1 and 2 stability formation have yet to be elucidated [77,78,79]. What is certain is these heptad repeat domains within F1 aid in joining virus and cell membranes—and are critical to viral-host membrane fusion [74,80].

#### 2.2.7. Small Hydrophobic Protein (SH)

The MuV small hydrophobic (SH) protein is the smallest protein of the virus at 6 kD and 57 amino acids in length—and is named for both its size and abundance of hydrophobic residues [63]. SH is not widely conserved amongst paramyxoviruses neither in residues nor in its orientation within the virion. This lack of conservation, though, makes it a primary choice amongst epidemiological virologists in utilization for sequencing characterizations of strains and their genotypes [81,82]. However, its small size and low expression levels can make identification of circulating strains a challenge due to varying success of extraction of the SH nucleotide sequence in field samples [21].

While nonessential for virus replication, SH is involved in mediating host immune responses. Deletion of this protein has been found to increase NF-κB activation and apoptosis in L929 cells in vitro, and inhibition of tumor necrosis factor-alpha (TNF-α) [83]. Xu et al., 2011 discovered that when a recombinant MuV with a deleted SH (rMuVΔSH) is intracranially introduced in infant rats, the virus displays attenuation and reduced neurotoxicity compared to its wild-type MuV counterpart [84].

#### 2.2.8. Hemagglutinin-Neuraminidase Protein (HN)

The counterpart to F, MuV’s hemagglutinin-neuraminidase (HN) protein is a type II membrane protein—meaning its N-terminus is arranged towards the viral interior and the C-terminus is extracellular [85]—of 582 amino acids in length and 80 kD size. Much like other paramyxoviruses, the MuV HN globular head domain forms a six-bladed beta-propeller fold and is formed by two homodimers to make a single tetramer [85,86].

HN’s primary role is during viral entry, where it recognizes sialyated glycoconjugates and binds to sialic acid as a receptor on host cells [87,88,89,90]. Similarly to influenza, which has both hemagglutinin (HA) and neuraminidase (NA) glycoproteins on its viral surface separately, the combo-HN of MuV and its binding behaviors have been attributed to viral pathogenicity. The hemagglutinin-portion of MuV HN is responsible for binding to the host cell, and binds preferentially to trisaccharides with a α2,3-sialic acid linkages, which can be found in various tissues and organs throughout the body—providing an explanation for the viral tropism for glandular tissues and ability for the virus to disseminate within the host [86]. A mutation at E335K in the Urabe vaccine strain for MuV has been attributed to an increased affinity for α2,6-sialic acid linkages. These sialic acid linkages are found in increased abundance on human neuroblastoma cells, making this MuV strain have increased neurovirulence due to this singular HN mutation [91,92].

Much again like influenza viruses, the HN has enzymatic activity as a neuraminidase. The neuraminidase-portion of the MuV HN removes sialic acid groups from budding virions, assisting in viral release from the host cell. This neuraminidase activity by HN aids in preventing aggregation of budding virions and potential viral receptors, reducing the impact of limited viral spread [93].

Lastly, HN is essential for the activation of F. Fusion between the host cell and viral membranes relies on this HN interaction for completion [94].

As both an enzymatic glycoprotein and essential player in viral fusion and release, HN is a major target of host immune responses in MuV infection [95] and antibodies produced by vaccination [17]. There are antigenic differences between HN proteins of different MuV genotypes. Genotype A, of which the US vaccine strain Jeryl-Lynn belongs, appears to be antigenically distinct from genotypes B, C, D, and H—with B-cell epitopes varying amongst all these genotypes for HN based on monoclonal antibody studies [96,97,98,99]. It has also been found that mutations in residues 12–14 and 127–129 of MuV HN of Genotype G, which includes the most recent outbreak strains (e.g., Iowa 2006), results in a loss of N-glycosylation—but remains conserved in “older” strains of other genotypes [17,100]. The effect of these changes is unclear—but what is clear is that there is growing evidence that the epitopic changes in HN across genotypes does appear to influence B and T cell responses, including variations in neutralizing antibody profiles of patients.

#### 2.2.9. Large Protein (L)

The largest of all paramyxoviruses and MuV proteins is the large (L) protein at 2261 residues in length and 200 kD in size. As the last transcriptional unit of the MuV genome, it has the lowest expression levels in MuV-infected cells. L is responsible for the enzymatic actions of initiation, elongation, and termination of RNA synthesis and MuV genome replication. It also, along with P as a cofactor, comprises the viral RNA-dependent RNA-polymerase (vRdRp) complex [101]. L also is responsible for 5′ capping to the 3′-poly(A) tail of transcribed viral mRNA and methyltransferase activity [101,102,103,104].

Mutations within L can be catastrophic to viral replication processes—which can occur naturally due to the erroneous nature of RNA viruses and its large transcriptional unit. The catalyzation of RNA synthesis in replication and transcription requires the five enzymatic regions and six amino acid residue domains of L. These first of regions are the RdRp or polymerase region, denoted as residue Domains II and III which possesses a positively charged RNA binding site and an active site for phosphodiester bond formation, respectively [105,106]. The second region is the polyribonucleotidyl transferase domain (PRNTase), which is found in Domain V. The third region is the connector domain (CD). The fourth region is the methyltransferase domain (MTase), found in residue Domain VI [102,103,104,107]. The fifth region is the C-terminal domain. Domains I, II, and IV have unclear individual functions, but mutagenesis studies have suggested that these residue domains may be involved in the regulation of the functional switch from transcription to regulation priorities of the polymerase [108]. The core module of L is the RdRp and PRNTase regions, as they lead the charge in RNA synthesis and capping of the transcript [102,103,104,109]. As such, this region is highly conserved amongst paramyxoviruses and other negative stranded viruses. The CD-MTase-CTD regions acts more as an appendage to the RdRp-PRNTase spear header and have high structural diversity amongst viral members of its family [103,110,111,112,113,114]. Due to the lack of complete L structures being elucidated by cryo-EM, the relationship between CD-MTase-CTD conformations and RNA synthesis has yet to be fully elucidated.

For the vRdRp, the formation of the P-L complex is of utmost importance. The N-terminal of P binds to L and directs the docking of L to the NP-vRNA template for genome synthesis. L becomes uncoordinated and cannot successfully perform replication and transcription without its P cofactor, as its secure attachment to the NP-vRNA template is jeopardized [22,115,116,117,118]. The relationship between P and L and its essentialness has been documented in almost every paramyxovirus, including Sendai virus, parainfluenza virus 5, human parainfluenza virus 3, measles virus, and vesicular stomatitis virus [101].

As the cofactor of L, P’s oligomerization domain self-oligomerizes P monomers and dimers. It is this self-oligomerization of P that helps facilitate the attachment of P to the RdRp region of L to tether the polymerase to the nucleocapsid and RNA template [39,50,118,119,120]. Not only does the N-terminus of P play a role in L coordination and docking, but the oligomerization domain with P assembly and attachment to L, the C-terminus and its X-domain within this terminus specifically is what can bind both RdRp and the NP. This X domain in P_CTD_ shows high diversity amongst negative-stranded viruses, and MuV is the only virus where its P can bind to L within both the N- and C-termini [112].

Originally, it was thought that four P_OD_ regions assemble into a coiled-coil structure that the P tetramer “cartwheels” on the nucleocapsid during the advancement of the polymerase across the NP-vRNA genome. During this P rotation, the L that is anchored to the X-domain of P_CTD_ dissociates from the RdRp to allow for the X-domain from another P monomer within the tetramer to rebind to the L [121,122,123]. This advanced “dance” requires binding of all four X-domains of P_CTD_ to continue in iterative cycles—however, up to three X-domains of P_CTD_ monomers can be impaired and RNA synthesis can still be maintained at comparable bioactivity [123]. This discovery, along with MuV P’s uniqueness in that multiple domains interact with L, lead to the cryo-EM of the L-P complex and for Li et al., 2024 to propose a new model to accommodate the idea that the oligomeric P does not have to undergo rotation and proposes a “tunnel” formation of the RNA genome that runs from the RdRp-PRNTase to the CD-MTase-CTD as a transcriptional state with four P molecules assembling like a “kettle spout”. For MuV, it appears that the L binding to the X domain of P_CTD_ on the side of entry for the RNA is performed by the second P monomer of the tetramer formation (P2) and allows for “competing” X-domains under the “sliding model” assumption [124].

## 3. Mumps Virus Replication

### 3.1. Mumps Virus Replication Cycle

#### 3.1.1. Viral Entry

MuV entry into a host cell is referred to as membrane fusion and is a common pathway of entry for enveloped viruses and predominant for paramyxoviruses. When infecting a host, HN protein binds to sialic acid on a host cell surface to initiate the viral-to-host cell membrane attachment. Once HN attaches to a host cell receptor, a conformational change occurs within the F protein of F_0_ to F_1_ + F_2_. This conformational change leads to fusion of the viral and cell membranes [74,93]. Once the MuV envelope is fused to the cell surface, the helical nucleocapsid and MuV’s genetic material are released into the cytoplasm of the host cell [46].

#### 3.1.2. Viral Replication and Transcription

MuV proteins NP, P, and L form the viral replication complex—these proteins are packaged within the virion and are released into the cell cytosol following fusion of the viral membrane to the host cell surface. The ribonucleoprotein (RNP) complex, comprising the MuV RNA genome and NP, functions as a protective mechanism for the genome against degradation from host cell enzymes and selectively exposes the genetic template for viral replication via vRdRp binding. An upstream start and downstream end signal sequence mark the “start” and “stop” signals for genomic replication, and the intergenic regions between transcriptional units contain *cis*-acting signals for viral transcription of these units into mRNA transcripts [109]. These gene-end (GE), intergenic (IG), and gene-start (GS) signals also play a central regulatory role by controlling transcription termination, polyadenylation, polymerase falloff, and reinitiation efficiency—determinants that shape the classical transcriptional gradient across the genome [28,46,51]. For viral replication and transcription, transcription is thought to begin first with the genome replicated later on in the viral life cycle. However, the initiator of this switch by which vRdRp remains to be elucidated [125,126,127]. The current proposed mechanism is that phosphorylation levels of P reach a “critical mass” point to trigger the vRdRp functional switch, due to P’s known ability to interact with both the RNP and nascent NP and its functionality in coordinating L to the MuV genome for both processes [30,31,39,52].

The synthesis of all MuV mRNA transcripts is initiated by the 3′ leader sequence. During replication and transcription, the vRdRp moves down the genome and across intergenic regions—once the vRdRp receives a “stop” signal in the intergenic region, it also must receive the “start” signal at the sequential gene’s start site. If the polymerase does not reinitiate this process, the gene order within the MuV may be mismatched and viral protein amounts made in inappropriate proportions [128,129]. Like other paramyxoviruses, MuV exhibits a transcriptional gradient in which genes closest to the 3′ promoter (e.g., N and P) are transcribed at the highest levels, while more distal genes (e.g., SH, HN, and L) show progressively reduced mRNA abundance due to sequential attenuation at each GE–IG–GS junction. This gradient has been documented across *Paramyxoviridae* and reflects incomplete reinitiation of polymerase at downstream GS sites [1,28,46]. In Figure 2, for instance, the first gene transcribed is NP which is found in the largest amounts in the cell. The last gene transcribed is L and its corresponding translated protein (RdRp) is found in the lowest amounts in the cell during viral replication compared to other proteins.

The MuV RNA genome is negative-stranded, so it is first replicated by the production of a positive-sense (or anti-genomic) RNA sequences. The anti-genome is then replicated into negative-sense vRNA by the vRdRp at the 5′ trailer sequence and subsequently packaged into progeny virions. The RNP can hold both the negative- and positive-sense vRNA genomes created during this process simultaneously. However, only the encapsidated RNA can be transcribed, as in the RNA originally bound to the NP—making the MuV RNP exhibit anti-termination functions by forcing the vRdRp to ignore transcription signals in intergenic regions of the genomes when bound to the RNP to produce successful, full-length RNA genomes [46].

When it comes to NP, P, and L proteins’ functions in viral replication and transcription, the structure of these proteins is critical for their mechanisms. The NP of paramyxoviruses share similar genetic identities, but their structure and formation varies between different viral species. Different paramyxoviruses have different requirements for their NP units, such as the number of NPs required to form one full ring of the helical capsid, the width of a helical capsid unit, and the distance between the turns of each of the stacked NP rings [130]. The interaction of each NP unit and its ring and helical structure is highly nuanced, and small changes to this structure can have great impacts on the ability for the polymerase to access the RNA genome and the stability of the RNA. Further, the P of paramyxoviruses has high variability in their tertiary structures and which domains interact with NP and L. Kinases and phosphorylation levels of P also varies amongst the paramyxoviruses [47,48,51,131]. NP and P structure within paramyxoviruses, and MuV, has vast implications for viral production. The position-dependent transcriptional output governed by intergenic regulatory elements underscores why precise NP–P–L coordination is essential: structural constraints that alter polymerase engagement with GE–IG–GS motifs can directly affect the steepness of the transcriptional gradient and thus the stoichiometry of viral structural proteins required for efficient virion assembly [31,51,107].

#### 3.1.3. Viral Assembly and Budding

To make more progeny virions, a MuV within a host cell must produce viral genome copies and viral proteins, assemble virions, and bud from the cell. Freshly synthesized RNA genomes are encapsidated by NPs produced and “floating” in the host cell cytoplasm, where it then associates with additional P-L complexes. mRNAs in tandem with this process are translated into viral proteins that are transported via the exocytic pathway to the inner folds of the host cell plasma membrane. At this interface, M will bind to the NP encapsidating new RNA genome, the cytoplasmic tails of new F and HN proteins, and organizes these proteins along the inner membrane of the cell. M, NP, and F have been shown to be essential players for efficient viral assembly and virion production. M is the conductor of this assembly process of progeny virions and their budding via the linkage of viral envelope to the RNP for incorporation into the new virus particle [65,132]. F contributes to virion egress, while HN acts to prevent self-aggregation of virions on their way out of the cell [74,87,94,133]. F and HN are also responsible for the “hallmark” cell-to-cell fusion seen both in tissue culture and pathologically, referred to as syncytia formation. Paramyxoviruses through F and HN interactions on the inside of the cell surface along the plasma membrane encourage syncytia formation, or the fusion of multiple host cells binding to one another to share organelles and contribute to providing multiple “viral factories” [73,94].

### 3.2. Viral Protein Factors Affecting the Viral Replication Cycle

The nucleoprotein, matrix protein, and defective interfering (DI) particles are some of the viral and host-interacting elements that affect the Mumps virus’s (MuV) replication cycle. To encapsulate the viral genome and form the ribonucleoprotein (RNP) complex—which acts as the template for RNA synthesis by the viral polymerase—the nucleoprotein (N) is necessary. This interaction controls the accessibility of viral RNA during transcription and replication in addition to protecting it. Viral replication can be severely hampered by changes to the structure of the N protein or its ability to bind RNA. Defective interfering particles, which are shortened genomes created during error-prone replication, also influence MuV replication. These DI genomes suppress the production of infectious viruses and may be involved in viral attenuation or persistence by competing with full-length genomes for replication and packaging machinery [65]. The matrix (M) protein is another important component that bridges the gap between the host cell membrane and the nucleocapsid to promote virion assembly and budding. Late domains in the M protein attract the host ESCRT machinery, facilitating virion release and membrane scission. Viral propagation efficiency can be limited, and virus release can be hindered by mutations in the M protein or its late domain motifs [66,67,68]. These elements work together to tightly control the MuV replication cycle’s dynamics, efficiency, and results.

#### 3.2.1. Nucleoprotein—The Role of Structure and Function

While NP is one of the three essential proteins for paramyxovirus replication, the protein’s morphology exhibits commonalities and differences between different members of this viral family. The MuV NP complex was characterized by using an *E. coli* expression system by the work of Richard Cox in 2009. It was found that when NP was co-expressed with P, empty NP-RNA rings formed with precisely 13 NP units. These rings did not require the presence of genomic RNA to form their structure. Once RNA was incorporated into the expression system, NP rings encapsidated precisely 78 nucleotides of the genome—which matched the expected size required to maintain the paramyxovirus “Rule-of-Six” principle by which the number of nucleotides within the viral genome is divisible by six. Cryo-electron microscopy (cryo-EM) of the purified NPs revealed a traditional herringbone structure common to many paramyxoviruses in culture [26].

**Figure 5:**
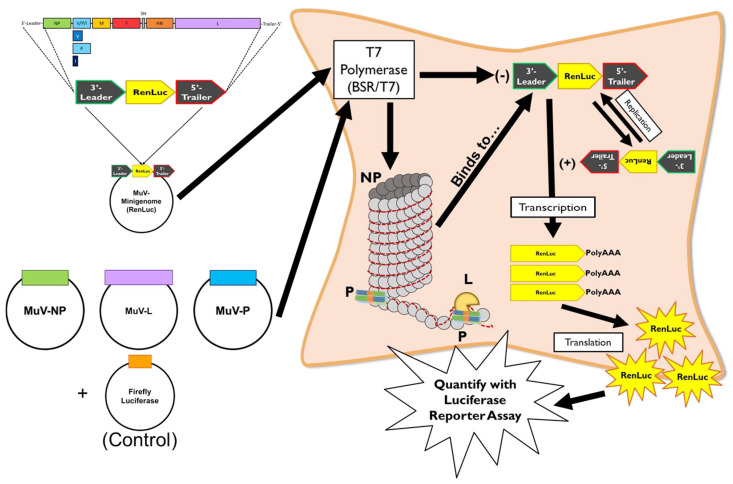
Mumps virus minigenome replication system. This figure represents the minigenome system used for evaluating the effects of the MuV replication proteins NP, L, and P on MuV replication. The 3′ leader and 5′ trailer sequences of the MuV genome are cloned into a pCAGGS plasmid, with a *Renilla* Luciferase expression gene between the leader and trailer. This plasmid is referred to as a “minigenome plasmid”, as it includes the leader and trailer sequence of the MuV full-length genome with a *Renilla* Luciferase protein sequence in-between. The MuV minigenome plasmid and pCAGGS plasmids encoding for MuV NP, L, and P are transfected, along with a Firefly Luciferase control pCAGGS plasmid, into BSRT7 cells. As BSRT7 cells express T7 polymerase, the T7 promoter in the pCAGGS plasmids is recognized and NP, P, and L proteins are transcribed. The NP, P, and L recognize the leader sequence of the minigenome plasmid and begin transcribing *Renilla* Luciferase. Thus, the more *Renilla* Luciferase that is detected by the luciferase assay once the cells are lysed, the more “viral” replication that has occurred. As the minigenome plasmid also expresses a T7 promoter, the Firefly Luciferase plasmid that has a T7 promoter is used as a control to compare “basal” cell level expression of Firefly Luciferase to that of *Renilla*, which would include both T7-based and NP, P, and L-based expression. Adapted from Risalvato et al., 2021 ©, with permission [134].

Cox et al., 2009 [26], to better understand the dynamics of the NP-P interaction, co-transfected RNP components to visualize their interactions. It was found that only when the P_CTD_ was expressed along with NO was there minimal observable changes in the NP structure—leading to the hypothesis that the P_CTD_ plays a key role in NP stabilization for the docking of L to the NP-RNA template. When the NP structure uncoiled to reveal the RNA template for replication, it was noted that the P_NTD_ was expressed along with the NP—suggesting the P_NTD_ playing a key role in the vRdRp’s ability to access the RNA template during genomic synthesis. Using a minigenome system (Figure 5), an in vitro process by which the three proteins NP, P, and L and their effects and mechanisms within paramyxovirus replication can be studied closely and quantified, revealed that both the P_NTD_ and P_CTD_ are required components for successful vRNA synthesis for MuV [33,38].

The structure of NP has only recently been crystallized by Shan et al., 2021, but the PIV5 NP structure has the strongest genetic and structural similarities to MuV’s NP at approximately 64% genetic similarity and has proven useful for comparative predictive analysis and broadening the understanding of how MuV NP’s structure affects replicative functions [34]. This publication has enabled a more detailed structural analysis and functional prediction of the MuV nucleoprotein (NP). Notably, amino acid residues 180–202 have been identified as a potential ‘hinge’ region that may transiently open to expose the viral RNA genome, allowing access by the viral RNA-dependent RNA polymerase (vRdRp) [34,50]. A comparable region is present in the NP of PIV5, although key differences in amino acid residues—particularly at positions 185, 197, and 200—may influence the structural stability and function of this hinge. Despite these predictions, the functional roles of these residues in MuV have not yet been validated experimentally [134,135]. Risalvato et al., 2025 [135], used cryo-EM imagery and overlays to predict regions and mutations of interest between MuV and PIV5 NP, altering critical residues of difference in the MuV NP to those represented in PIV5′s NP. The results showed that residue 200 within the hinge region required to access the RNA template by the vRdRp was detrimental to viral replication and viability. A mutation at residue 185, also in the hinge region, resulted in viability virus but a delay in growth kinetics compared to wild-type virus—hinting that mutating this site led to slower replication kinetics at the flexible region. A mutation in the hinge region at residue 197, critical for forming the RNA binding groove and a proposed site for P_CTD_ binding, was viable but displayed decreased viral growth kinetics at high viral loads in tissue culture [135].

Additionally, because the cryo-EM structure of the MuV nucleoprotein (NP) lacked bound RNA, unlike the PIV5 NP structure where the RNA genome was crystallized, it remains unclear whether the observed residue differences are involved in interactions with RNA or the viral polymerase (L protein). However, the absence of RNA in the MuV NP structure revealed an open RNA-binding groove, which is thought to facilitate access by the viral RNA-dependent RNA polymerase (vRdRp) during RNA synthesis [136]. The C-terminal tail of NP is intrinsically disordered, and although this region was not resolved in the structural model, it was shown to be nonessential for nucleocapsid assembly [30,33]. Despite not contributing to structural formation, deletion of the NP C-terminal tail abolishes activity in the minigenome replication system, indicating that this region is critical for RNA synthesis, potentially through direct interaction with the RNA genome or with the vRdRp [26,136].

Modifying the structure of the MuV nucleoprotein (NP) can have broad functional consequences, as NP serves multiple critical roles throughout the viral life cycle. These include protecting the viral RNA (vRNA) genome, exposing the RNA template for transcription by the viral RNA-dependent RNA polymerase (vRdRp), stabilizing the antigenome, facilitating anti-termination during genome replication, encapsidating newly synthesized vRNA, and supporting virion assembly. NP also assists in the proper localization and chaperoning of viral components to the host cell surface for budding. Understanding how structural changes in NP impact these diverse functions would enhance our knowledge of MuV replication and may reveal novel targets for antiviral therapeutics.

#### 3.2.2. P Structural Impacts on Viral Replicative Functions

The canonical NP-binding domain (NBD) found in other Paramyxoviridae family members is consistent with this region [112]. Nevertheless, MuV P can interact with NP via more than just its C-terminus. The N-terminal domain may also aid in NP binding, according to experimental evidence [119]. According to analyses using electron microscopy, the P_NTD_ helps to relax the nucleocapsid’s helical structure. In viral minigenome assays, this conformational shift has been linked to higher RNA synthesis efficiency and improves accessibility of the encapsidated genome [135].

Structural studies have delineated the oligomerization domain of MuV P as residues 213 to 277. Unlike other paramyxoviruses, the MuV P tetramer features an unusual assembly in which two parallel α-helical dimers align in an anti-parallel fashion. This arrangement, which is believed to support both structural stability and biological function, results in two N-termini and two C-termini at opposite ends of the oligomer [119]. Binding to NP requires the P_CTD_, which is made up of amino acids 343 through 391 and corresponds to a conserved NP-binding region (NBD) shared by all paramyxovirus P proteins [112]. Interestingly, the C-terminus is not the only location where this interaction occurs. There is evidence that the N-terminal domain of the P protein also participates in NP binding [119]. Minigenome replication assays and electron microscopy visualization has demonstrated that this N-terminal region can unwind the helical structure of the viral nucleocapsid, a conformational change that stimulates increased viral RNA synthesis [38].

Given that both the N-terminal and C-terminal regions of the mumps virus (MuV) phosphoprotein (P) can interact with the nucleoprotein (NP), it is possible that the unique tetrameric architecture of P facilitates or enhances this dual binding capacity. Functional analyses using a minigenome system have shown that neither the N- nor C-terminal domains of MuV P alone are sufficient to support viral RNA replication in the absence of the oligomerization domain. Replication activity was restored, however, when both terminal domains were co-expressed with the oligomerization domain present, suggesting that the oligomerization domain is necessary to allow for structural or cooperative complementation between P fragments [38,119]. This study will delve deeper into the phenomenon of structural interdependence, also known as trans-complementation, which highlights the crucial role of oligomeric integrity in P-mediated replication.

#### 3.2.3. Defective Interfering Particles and Genomes

Defective interfering particles (DIPs) are viral particles that, while containing standard structural proteins, harbor only partial segments of the viral genome and are capable of replication only in the presence of a helper virus. Notably, their interference was shown to be highly specific, targeting the intracellular replication processes of the homologous, fully functional virus. DIPs have been linked to the development of persistent infections both in vitro and in vivo. For instance, experimental infections in mice demonstrated enhanced survival in the presence of DIPs, likely due to their capacity to stimulate interferon production [137,138,139,140,141,142,143,144,145]. This phenomenon had been observed in a wide range of RNA viruses—such as poliovirus, rabies, measles, human parainfluenza virus, Semliki Forest virus, Ebola virus, and respiratory syncytial virus (RSV)—all of which were found to generate DIPs when propagated under high MOI conditions [138,146,147,148,149].

DIPs appear characteristically similar if not equivalent to virus particles morphologically, but harbor genomes riddled with deletions or misfolds—referred to as defective viral genomes (DVGs). These DVGs either get packaged and released, inhibiting the replication cycle success in the next host cell or interfering with replication cycle dynamics in the initial host cell where they were generated. DVGs have high heterogenicity when considering the type, structure, deletion location(s), and genomic sizes. What is consistent, however, is that DVGs have the initiation and termination sequences so that they can be recognized and replicated by viral polymerase. Due to the error-prone nature of the RdRp, RNA viruses have a high intrinsic ability to naturally generate DVGs and DIPs. Additionally, mutations and structural changes in replication proteins can lead to mismatches of replication complexes and even “misprints” of genomic transcripts and accumulation of DVGs. DVGs are often packaged without clear differentiation between defective and intact viral genome copies, and as such these DIPS harboring DVGs can bud and enter a new host cell as well as a standard virus particle. However, DVGs possess a replicative advantage over intact genomes given that they are often shorter and preferentially replicated by viral polymerases over full length genomes, predominating viral replication patterns and acting as a pseudo-parasite in competition with true viral genomes. Additionally, if parasitizing off replication machinery was not inhibitory enough, the overflow of short viral genomic transcripts can stimulate innate immune responses in the cell to trigger antiviral pathways—further jeopardizing viral success in infection [138,150,151].

DVGs can form when viral polymerases lose their processivity and “fall off” the genomic template and reattach elsewhere along the genome to complete the viral genome replication cycle. Since the NP of paramyxoviruses, including MuV, can hold both the sense and anti-sense genomes during replication—this effect is often exacerbated and can lead to high heterogenicity and complexity of DVGs. DVGs are often categorized as three main forms: deletion, copyback, and snapbacks [152,153]. Deletion DVGs are created when the viral polymerase detaches from the genome template and reattaches downstream. These deletion DVGs share 3′ and 5′ ends with their full-length viral genome counterparts. Copyback DVGs are created when the viral polymerase detaches but then reattaches to the antigenome strand of RNA it was producing, which synthesizes a sequence of complementary nucleotides on the antigenome strand to the 5′ vRNA end. This rejoining occurs at a nonhomologous region, creating a nonhomologous loop structure flanked by complementary ends. Lastly, snapback DVGs form in a similar fashion to copyback DVGs—but do not have the “loop” formation seen in copybacks and is almost entirely a complementary genome with as little as one noncomplementary nucleotide as the breakpoint. For this tightened genomic structure to occur, the viral polymerase falls off the template at this narrow breakpoint and reattaches quickly to the nascent strand at a nearby rejoin point to make a copyback-esque DVG but without the large loop structure [148,151,154,155,156,157,158]. A fourth DVG, called the mosaic DVG, can sometimes occur and is described as a DVG that is a mixture of any combination of deletion, copyback, and/or snapback components.

Risalvato et al., 2025 discovered that three mutations within a region denoted as “Top” due to their orientation within the NP subunit—N63G, P139D, and A197Q—showed increased DIP production that inhibited viral production at both high and low MOIs. It is hypothesized here that the “Top” domain of the MuV NP plays a role in the ability for the polymerase complex to stay attached to the RNA template during elongation. Changes to this site increased vRdRp instability, likely due to the “Top” domain failing within the N-terminal region of MuV NP critical for the formation of the “RNA groove” where the template lies for vRdRp access, and as such interactions between this domain and vRdRp are required for exposure of the RNA template and maintenance of the attachment of the polymerase during translocation of the complex [135].

#### 3.2.4. Matrix Protein and Late Domains

The M protein of mumps virus (MuV) is essential for orchestrating virion assembly and coordinating the viral proteins in close proximity at the plasma membrane for budding. M protein acts as a bridging element between the outer envelope glycoproteins and inner RNP complex proteins, regulating the budding process of progeny virions to ensure each virion contains a nucleocapsid with a genome for successful completion and emergency of infectious virus. In many enveloped RNA viruses, such as retroviruses and filoviruses, the efficiency of this process depends on the presence of so-called late (L) domains, which are conserved sequence motifs such as PT/SAP, PPXY, and YPXL that facilitate recruitment of host endosomal sorting complex required for transport (ESCRT) proteins. Host ESCRT proteins assist in the mediation of membrane deformation essential for the detachment of virions from the plasma membrane. Mumps virus budding is ESCRT-dependent, despite lacking classical L domains in the matrix protein. Li et al., 2009 showed that the production of virus-like particles (VLPs) from cells co-expressing MuV M and NPs is significantly decreased when dominant-negative mutants of ESCRT-associated proteins, such as VPS4AEQ and CHMP4bΔC, are overexpressed [65]. This implies that MuV engages in indirect interactions with the ESCRT machinery, either through adaptor host proteins or cryptic or non-canonical M protein sequences that function similarly to L domains. Because other paramyxoviruses, such as Sendai virus and PIV5, also lack classical L domains but rely on ESCRT components for budding, this suggests a broader family-level strategy involving alternative ESCRT recruitment pathways [159,160]. MuV’s reliance on odd motifs or host co-factors may make it less efficient at budding or more vulnerable to mutations or cellular restriction factors that block these alternative pathways [68,132].

In addition to its function in membrane scission, the MuV M protein must interact with NP to guarantee that viral genomes are packaged correctly. This interaction is unique to a specific sequence and is calibrated precisely. Li et al., 2016 found that a highly conserved tripeptide motif (DWD) near the NP C-terminus is essential for its interaction with M. In addition to preventing NP incorporation into budding sites, the central tryptophan mutation (W to L; resulting in a DLD motif) significantly decreased the production of VLP. This disruption indicates that a direct M–NP interaction is essential for initiating or sustaining virion assembly, rather than being merely structural. If this interaction is lost, particles that do not cause disease but have a damaged genome may form. The DWD motif is also found in many paramyxoviruses, which suggests that this interaction is an important part of how viruses in this family come together and could be a good target for broad-spectrum antivirals [161].

In the grand scheme of viral replication, the lack of canonical L domains might lead to MuV particles being released less effectively or at a slower pace, particularly under cellular stress or in the presence of host restriction factors induced by interferon. It has been demonstrated that in other viruses deficient in robust L domains, particle release is more vulnerable to inhibition by host responses or mutations in host machinery. Moreover, if MuV depends on a narrow set of host interactions to compensate for the missing L domains, it could represent a replication bottleneck in vivo—contributing to variation in virulence, persistence, or tissue tropism. The bottleneck could also explain MuV’s occasional failure to establish productive infections in certain cell types or during vaccine attenuation, where viral replication may be limited by inefficient budding.

### 3.3. The Role of Host-Factor Protein Regulation in Mumps Virus Replication

#### 3.3.1. P Phosphorylation Affecting MuV Replicative Functions

MuV P, like other members of the Paramyxoviridae family, is crucial for scaffolding the formation of a functional viral RNA-dependent RNA polymerase (vRdRp) complex with the large (L) protein and nucleoprotein (NP). For transcription and replication to proceed with processivity and fidelity, NP-P interactions must direct the vRdRp to encapsidated viral RNA [39,50]. According to structural and biochemical studies, P binds to the disordered C-terminal tail of NP to prevent premature NP self-assembly and ensure that only newly synthesized viral RNA is encapsidated. Similar viruses such as the Sendai virus and measles share this mechanism [33,162,163]. Replication kinetics and accuracy are thus directly impacted by any modification of the NP–P interaction.

One of the most important regulators of P protein function is phosphorylation. Phosphorylation at specific serine and threonine residues in the intrinsically disordered N-terminal domain of the MuV P protein is required for its ability to mediate NP binding and to activate L polymerase function [47,51]. Phosphorylation increases P’s conformational flexibility and may encourage the polymerase complex to assemble into higher-order oligomeric forms. P protein phosphorylation in paramyxoviruses has been linked to host kinases, specifically casein kinase II (CKII) and protein kinase C (PKC) [164,165]. Defective P phosphorylation, disturbed NP–P complex formation, and reduced viral replication result from inhibiting these kinases in tissue culture—indicating that the virus has appropriated these enzymes to optimize its replication machinery.

Dynamic studies of phosphorylation in the context of MuV infection have revealed that the temporal regulation of P phosphorylation may act as a molecular switch for toggling between transcription and replication [40,48]. In the early stages of viral infection, when MuV mRNA synthesis is prioritized, lower levels of phosphorylation may be sufficient to stabilize the P–NP interaction needed to initiate transcription. However, as infection worsens and genome replication becomes crucial, the hyperphosphorylation of P may result in conformational changes that shift the polymerase complex into a replication-competent state. The virus can temporally control its life cycle through this phospho-switch mechanism without the need for extra proteins or outside signals.

A key factor in regulating viral replication efficiency and replication complex assembly is host-mediated phosphorylation of the MuV P. The cellular kinase RPS6KB1 was recently discovered to be a negative regulator of MuV replication by Briggs et al., 2020 [49]. Viral RNA synthesis requires the formation of productive P–NP complexes, which can be inhibited by RPS6KB1′s phosphorylation of the P protein. Infected cells and minigenome systems in this work showed a marked increase in both viral genome replication and mRNA transcription when RPS6KB1 function was lost via siRNA knockdown, pharmacologic inhibition, or CRISPR-mediated knockout. This suggests that RPS6KB1 acts as a phosphorylation-based “brake” on viral replication processes.

MuV P can also be phosphorylated by Polo-like kinase 1 (PLK1), but this regulatory function has the opposite effect. By encouraging P’s binding to L, PLK1 promotes the formation and functionality of the vRdRp complex, aiding in viral replication [48]. These results illustrate the idea that conflicting results can occur when different host kinases phosphorylate the same viral protein. For example, PLK1-mediated phosphorylation promotes the formation of an efficient replication complex, optimizing RNA synthesis, whereas RPS6KB1-mediated phosphorylation inhibits replication, possibly as an innate antiviral mechanism. In addition to providing insight into how viruses take advantage of or avoid host control, knowledge of the kinase-specific regulation of viral protein interactions also suggests potential targets for antiviral therapy.

Other host kinases, including CK2 and various PKC isoforms, have also been shown to phosphorylate the MuV P, and these modifications influence its functional interactions with NP and L. Phosphorylation of P can modulate its ability to stabilize NP^0^, ensuring proper nucleocapsid assembly, and also regulates the switch of the polymerase from transcription to replication [119]. By controlling these processes, host kinases effectively tune the efficiency and timing of viral RNA synthesis, highlighting another layer of host-mediated regulation of MuV replication.

Given the centrality of phosphorylation in controlling P function, targeting these post-translational modifications presents a promising avenue for antiviral intervention. Yabukarski et al., 2020 showed that it may be possible to hinder MuV replication while preserving host cell machinery through the utilization of small molecules that selectively block the host kinases most involved in P phosphorylation or competitively disrupt NP-P or P-L interactions via imitation of presently phosphorylated or unphosphorylated protein states [166]. These regulatory processes may also aid in the attenuation of vaccine strains, as modified P phosphorylation kinetics may lessen pathogenicity and replication efficiency in vivo.

#### 3.3.2. Phosphorylation of Other Mumps Proteins

The MuV nucleocapsid protein (NP) is phosphorylated at multiple locations, with serine 439 (S439) emerging as a crucial regulatory residue, according to a seminal study by Zengal et al., 2015 [40]. They identified nine phosphorylated residues and produced alanine mutants to evaluate their functional impact using radiolabeling and LC-MS analysis in infected cells. Interestingly, the S439A mutant showed a phosphorylation loss of >90%. Surprisingly, when compared to wild-type NP [40], viral minigenome assays revealed that this S439A substitution increased RNA synthesis and viral protein expression during early infection. These results suggest that phosphorylation at S439 functions as a molecular brake, restricting mRNA transcription and early rounds of RNA replication [40]. It probably acts as a temporal checkpoint to maintain a balance between transcription and replication.

The crucial role of S439 was confirmed by additional in vivo tests using recombinant mumps virus. The idea that phosphorylation of S439 regulates replication dynamics is further supported by the observation that viruses carrying the S439A mutation exhibited increased viral protein output and accelerated early genome replication [40]. Interestingly, dephosphorylation at this location may interfere with downstream steps of the viral cycle, including NP assembly or genome encapsidation, despite initially increasing replication machinery activity. According to this observation, S439 phosphorylation guarantees controlled progression throughout the replication/transcription continuum, avoiding excessive or premature viral RNA synthesis that could otherwise result in aberrant genome processing or early immunological detection.

Zengel et al.’s [40] research establishes S439 as a crucial regulatory phosphorylation site within MuV NP, acting as a switch that regulates the intensity of early replication. One intriguing potential strategy for antiviral treatment could be to target the kinase that phosphorylates S439 or imitate its phosphorylated state as a method to compromise MuV replication integrity and hinder effective virion production.

#### 3.3.3. Host Chaperone Proteins in the Regulation of Mumps Virus Replication

Host heat shock proteins (Hsp) are known to assist other proteins in folding correctly, correct misfolded proteins, and prevent protein aggregates as molecular chaperones. Hsp70 and Hsp90 are critical to the replication cycles of many negative-sense RNA viruses, including those in the Paramyxoviridae family, by directly interacting with viral structural proteins to regulate nucleocapsid assembly, polymerase activity, and overall replication efficiency. Although there is less research on MuV than on measles, Sendai, or Nipah viruses, these chaperone mechanisms appear to be conserved throughout the family and are strongly implicated in MuV replication [1,28].

Hsp70 has been shown to bind directly to the nucleoprotein (NP) of several paramyxoviruses, facilitating proper NP folding, stabilization of the NP–RNA complex, and prevention of nonproductive NP aggregation during nucleocapsid formation [167]. For viruses such as measles and Nipah, Hsp70 recruits additional NP monomers to the growing nucleocapsid and enhances the efficiency of polymerase loading onto the NP-RNA template. Similar interactions have been observed for MuV NP in co-immunoprecipitation assays, suggesting that Hsp70 may assist in stabilizing MuV RNP architecture and promoting replicative polymerase engagement [168].

By helping to stabilize the L polymerase and its related phosphoprotein (P) cofactor, Hsp90 also plays a crucial regulatory role in paramyxovirus replication [169]. Inhibition of Hsp90 causes L to quickly degrade or misfold in several paramyxoviruses, which effectively stops viral transcription and replication. Hsp90 may promote the formation of L–P complexes and seems to keep L in a replication-competent conformation. These results strongly imply that Hsp90 is similarly necessary for preserving MuV polymerase stability and activity, given the structural similarity of MuV L and P to those of related paramyxoviruses [169,170].

Hsp70 and Hsp90 function as essential host chaperones that directly support multiple stages of viral RNA synthesis through their interactions with NP, P, and L. Their functions underscore the dependence of MuV on host protein-folding machinery and highlight potential therapeutic targets for modulating viral replication.

## 4. Host Immune Response to Mumps Virus

### 4.1. Innate Immune Recognition

#### 4.1.1. Pattern Recognition Receptors and Interferon Signaling

The host cell’s cytoplasmic pattern recognition receptors (PRRs), which can identify a range of distinct “foreign” nucleic acid constructs, including negative-stranded RNA, identify a virion when it enters the cell. RIG-I-like receptors (RLRs), including melanoma differentiation-associated protein 5 (MDA5) and retinoic acid-inducible gene I (RIG-I), are essential for detecting viral RNA inside cells. MDA5 recognizes the longer double-stranded RNA structures that form during viral replication, whereas RIG-1 detects panhandle structures or short double-stranded RNA that contains 5′-triphosphate and is found in MuV genomes. These PRRs trigger a downstream signaling cascade that activates the transcription factors IRF3, IRF7, and NF-kB when these viral RNA “trademarks” are present [167]. These transcription factors help to “turn on” genetic transcripts within the host cell to induce the production of type I interferons (IFN-α and β) and other pro-inflammatory cytokines.

#### 4.1.2. Type I Interferon Responses and Mumps Virus Antagonism

The type I interferon response is triggered by MuV infection and plays a critical role in the establishment of the “antiviral state” of an infected host cell. IFN-α and β, when secreted, bind to the IFN-α/β receptor (IFNAR) to initiate the JAK-STAT signaling cascade. This cascade induces the phosphorylation of the STAT1/2 genes within the host cell, forming an ISGF3 transcription complex. This complex translocates to the nucleus to activate the transcription of interferon-stimulated genes (ISGs) capable of exerting more diverse antiviral functions such as inhibiting viral replication, degrading viral RNA, and slowing or shutting down cellular metabolism [171]. However, MuV has evolved mechanisms to evade host immunity. For example, the MuV V protein binds to MDA5, inhibiting its signaling, and targets STAT1 for proteasomal degradation. These actions make MuV V protein capable of suppressing both IFN production and downstream signaling responsible for inducing the antiviral cell state [55,172]. Furthermore, the SH protein has been documented to dampen the host immune responses, but its specific roles are unclear—what is known, though, is that deletion of SH from the MuV leads to attenuated viral growth in vitro and in vivo, implicating SH’s role in innate immune responses [84,173]. The innate immune antagonism enables MuV to replicate more efficiently and contributes to viral persistence and pathogenicity.

#### 4.1.3. Innate Immune Responses with Mumps Tissue Tropism

MuV primarily replicates in the upper respiratory tract, but it can also be neurotropic, leading to more severe complications like deafness, meningitis, or encephalitis. The airway epithelium is usually the site of the first infection, where MuV replicates after entering cells through sialic acid receptors [17]. Although MuV proteins like V protein can block IFN signaling to enable effective replication and dissemination in the host, these cells do react to viral infection by activating PRRs, which starts type I IFN responses to stop viral spread [56,172]. However, in the earliest stages of infection, MuV further dampens antiviral defenses through the V protein’s ability to inhibit MDA5 signaling and interfere with STAT1/STAT2 phosphorylation, resulting in reduced type I interferon induction even in cells that typically mount strong antiviral responses, such as microglia. This early blunting of IFN signaling enables MuV to persist long enough to disseminate systemically [55,174]. MuV can penetrate the blood–brain barrier and infect neural tissues, which are frequently referred to as “immune privileged” sites, after viremia has occurred. Compared to their counterparts in peripheral tissues, neurons and ependymal cells in the central nervous system typically have fewer innate immune defenses, making them prime targets for MuV infection. The mechanism of CNS entry is not fully resolved, but current evidence suggests that MuV may cross the blood–brain barrier either by infecting or altering the function of vascular endothelial cells—thereby increasing local permeability—or through a “Trojan horse” mechanism in which infected mononuclear cells traffic the virus across the barrier during viremia [1,6]. Despite strong systemic immune activation, MuV meningitis or encephalitis may develop because of the virus’s defenses against the antiviral cellular state and the immune-privileged environment of the central nervous system [174]. Together, these features illustrate how MuV evades early antiviral signaling while exploiting structural and immunological vulnerabilities of the CNS. MuV’s ability to take advantage of tissue-specific variations in innate immunity for both transmission and pathogenesis is highlighted by this dual tropism.

MuV infection can also spread to the male reproductive tract, causing one of the more well-known complications, orchitis, which can impair fertility and, in some cases, lead to long-term subfertility. Despite this, the immune responses that occur in reproductive tissues following MuV infection are not fully understood. In a 2016 study, Wu and colleagues found that MuV triggers innate immune responses in Sertoli cells, which support sperm development, and Leydig cells, which produce testosterone, in mice. These responses were mediated through TLR2 and RIG-I signaling pathways and led to the production of inflammatory mediators like TNF-α, IL-6, MCP-1, CXCL10, and type I interferons. Interestingly, male germ cells did not show the same kind of response. Sertoli cells produced more proinflammatory cytokines and chemokines than Leydig cells but had lower type I interferon output. When TLR2 or RIG-I were knocked down, the immune response was significantly reduced in both cell types. The researchers also found that MuV infection reduced testosterone production in Leydig cells—a notable observation, given the well-documented inverse relationship between testosterone levels and immune function [175]. These findings highlight how MuV can disrupt immune-privileged tissues like the testes and may help explain its link to reproductive complications.

### 4.2. Adaptive Immunity

#### 4.2.1. Humoral Responses: Neutralizing Antibodies

Neutralizing antibodies (nAbs) that target the MuV F and HN membrane glycoproteins are currently the primary diagnostic correlate of protection. Population studies have established genotype-specific neutralizing thresholds: a titer of ≥16–20 against genotype G (current circulating wild-type genotype in North America) is correlated with protective immunity [176]. However, military personnel data suggests that a Jeryl-Lynn vaccine (genotype A) neutralization above 41 is predictive of MuV vaccinated persons being protected against genotype G [177]. Additionally, immune escape by wild-type genotypes has been documented in outbreaks among vaccinated populations. Sera from naturally infected or vaccinated-infected individuals have shown significantly higher neutralization capabilities of genotype G strains compared to individuals who have only been vaccinated [178].

Total serum anit-MuV IgG measured by enzyme-linked immunosorbent assay (ELISA) is poorly correlative with neutralization activity of antibodies due to differential responses to non-neutralizing antigens such as NP. Plaque reduction neutralization titers (PRNT) remain the diagnostic gold standard for the evaluation of functional humoral immunity [179].

Long-term sero-epidemiological data has shown that humoral immunity that is either vaccine-elicited or infection-acquired is remarkably durable—one study revealed that 90% of the subjects remained seropositive for MuV IgG over decades with an estimated antibody half-life of over 500 years. However, periodic boosts from asymptomatic re-exposures into the population were noted, and their effect on this durability is unknown [180]. Renewed outbreaks nevertheless have continued with the emergence of new genotypes, particularly among young adults. It has been hypothesized that this is driven by waning genotype-specific neutralizing capacity despite the total preserved MuV IgG levels within the vaccinated population. College-aged individuals have displayed a six-fold lower neutralizing GMT against genotype G compared to the vaccine strain despite a high seropositivity for general MuV nAb [181].

Humoral immunity to MuV involves complex interplay of both total and nAb responses. Protection is not just contingent on seropositivity, but on the presence of functional nAb titers. Furthermore, strain-specific nAbs play a strong role in the functionality of immune protection against circulating genotypes. While long-lived serum IgG against MuV has been documented, the rising incidence of genotype G outbreaks in the modern era among vaccinees highlights the critical growing need for enhanced vaccination strategies such as genotype-matched boosters or assays focused on functional over total antibody responses.

#### 4.2.2. Cellular Immunity: T Cell Responses and Memory Formation

As with most viral infections, CD8^+^ cytotoxic T lymphocytes (CTLs) are essential in controlling MuV infection. A recent study by Kaaijk et al. used mass spectrometry and immunological assays to identify over forty naturally processed MuV-derived HLA class I epitopes. Five of these identified epitopes were restricted to HLA-A01:01 and HLA-B07:02 and elicited strong CTL responses with cytotoxic activity in naturally infected individuals. However, these polyfunctional CTL responses are largely absent in recently vaccinated individuals, displaying a substantial difference in the cellular responses elicited by live virus vs. vaccine exposure [179]. This could be in part to the high genetic differences between vaccine strain epitopes to wild-type MuV-elicited epitopes—genetic analyses comparing vaccine strain elicited epitopes to those from 462 circulating wild-type MuV genomes revealed that 78% of CTL epitope candidates had amino acid differences, and 43% exhibited reduced predicted HLA class I binding in circulating strains. This antigenic variability would likely impair cross-recognition of wild-type MuV by T cells induced by vaccination alone. An additional study found that naturally infected adults maintained these MuV-specific CTL responses for at least three years post-infection, whereas vaccinees exhibited a lower magnitude and polyfunctionality in their CTL repertoire. These polyfunctional CTL responses have also been marked with increased IFN-γ production and increased cytotoxic potential [182,183].

CD4^+^ helper T cells also contribute significantly to antiviral immunity through cytokine secretion and humoral support. It has been reported that vaccinated and naturally infected adults both produce CD4^+^ cells capable of IFN-γ secretion upon MuV antigen stimulation, but there was limited significant difference between treatment groups. However, what was found was a defined epitope in naturally infected groups from the MuV NP (N_110–124_; GTYRLIPNARANLTA) that was recognized by Th_1_-type CD4^+^ T cells across multiple HLA-DRB1 haplotypes, producing IFN-γ, TNF, IL-10, and other cytotoxic markers. This epitope highlighted the multifunctional character of CD4^+^ responses after natural infections [184].

The memory T cell phenotype and its durability has been characterized in naturally infected individuals. Longitudinal analysis of HLA-A2-restricted CTLs revealed a contraction of short-lived effector cells over nine months with an increased proportion of memory precursor and long-lived effector cells. This indicated stable, long-lived T cell memory responses in these individuals. However, vaccine-induced T cells appear less diverse and robust in their responses—one report found that up to 78% of the identified vaccine-recognized and induced epitopes exhibited amino acid variability in circulating wild-type MuV strains, which could undermine CTL recognition and cross reactivity [183].

In summary, natural MuV infection has been found to induce a robust, polyfunctional CD8^+^ and multifunctional CD4^+^ T cell response against a broad spectrum of MuV epitopes. These T cell populations have also been documented to form durable long-lasting memory subsets that likely contribute to life-long protective immunity. Vaccination, on the other hand, generally produces a weaker and less diverse T cell response with limited epitope coverage and reduced polyfunctionality—all factors that may contribute to decreased protection and vulnerability to wild-type MuV outbreaks in an ever-changing epidemiological landscape.

### 4.3. Immunogenicity Variation Across Genotypes

While MuV is classified into a singular serotype, there is significant antigenic variation existing amongst all genotypes, particularly between the Jeryl-Lynn vaccine strain (genotype A) and circulating wild types (most notably genotype G in North America). Epidemiological and serological investigations have cited reduced nAb titers against genotype G in vaccinated individuals, with vaccinated sera exhibiting approximately a 3.7-fold and 2.3-fold lower titer against genotype G’s HN and F proteins, respectively, when compared to the vaccine strain [173,176]. In contrast, persons infected with newer genotype G MuV strains mounted stronger responses to HN and F genotype G versus A. The antigenic mismatch occurring suggests that genotype A-based immunity does not confer equal cross-protection across genotypes. Complementary mouse studies using genotype-matched vaccine constructs (F and G) have shown enhanced nAb responses to genotype G and F strains—supporting the concept that genotype-specific boosters have restorative cross-neutralization capacity [173,185].

There have been notable differences in the divergence of B cell epitopes on HN and F proteins within genotype G viruses compared to Jeryl-Lynn vaccine strains. In the 2016 Arkansas outbreak, computational genomic and immunoinformatic predictions flagged differences in the predicted B cell epitopes in experimentally verified immunogenic regions, implicating the potential of genotype G immune escape within vaccinated populations [186]. Furthermore, the previously discussed study by Shaikh et al., 2024 cited genomic differences in HLA class I epitopes with high amino acid variation and reduced predicted HLA binding, suggesting the potential impacts on cellular immune responses and cross-reactivity [176]. However, laboratory neutralization assays have yielded differing conclusions with some reporting reduced cross-neutralization by vaccine-induced sera, and others finding no significant differences [186]. These findings underscore the complexity of immune escape phenomena of MuV.

The growing evidence of reduced immune cross-reactivity between currently circulating and vaccine MuV strains—particularly with genotype G—suggests that reformulating the existing vaccine or including a genotype-matched booster to aid in diverse antigenic profile recognition may be necessary to see improved immune coverage against MuV infection. Incorporation of conserved neutralizing epitopes within key viral proteins such as HN and F could broaden antibody responses and enhance cross-genotype protection. Additionally, rational epitope design accounting for high-variability regions may assist in the reduction in immune escape and help with future outbreak mitigation, even in highly vaccinated populations.

## 5. Epidemiological Trends and Outbreaks

MuV has demonstrated fluctuating epidemiological trends over recent decades, with periodic outbreaks occurring even in highly vaccinated populations. While the initial introduction of the MMR vaccine in the 1960s caused a significant reduction in the global incidence of disease, resurgences in the 21st century have been reported. These newer MuV cases have been interestingly occurring primarily among adolescent and young adults in close-contact settings such as universities and military barracks. Although MuV is considerably less transmissible than some other respiratory viruses, such as its measles virus counterpart in the MMR vaccine formulation, outbreaks have shown that transmission can occur through either casual or indirect respiratory contact. This indicates that prolonged or highly direct exposure is not always required for MuV transmission [186]. Combined genomic and epidemiologic analyses estimate the effective reproduction number (R_e_) of MuV to be approximately between 4 and 7, which is sufficient to sustain transmission within semi-closed or high-density populations [187].

These findings underscore that while MuV spreads less efficiently than other respiratory viruses, social mixing patterns and waning vaccine-induced immunity can create favorable conditions for outbreaks. These outbreaks are often attributed to the perceived waning of vaccine-derived immunity and the emergence of genotypic variants that may escape neutralization by vaccine-induced antibodies and limited immunological memory. Recent reviews have similarly emphasized that waning immunity to MuV, rather than solely antigenic shift of the virus, remains the predominant driver of MuV reemergence in highly vaccinated populations [188].

Despite high two-dose MMR coverage, these trends highlight the importance of ongoing surveillance, booster dose evaluation, and consideration of genotype-specific vaccine updates.

### 5.1. Global and Regional Case Trends

MuV infection was considered a nearly universal fact in early childhood before the advent of the MMR vaccine—with 90% seropositivity by age 15 in unvaccinated populations. Once the monovalent and later a two-dose MMR vaccination program was introduced between 1960 and 1990, respectively, many countries experienced a dramatic reduction in disease incidence [189,190]. Canada, for instance, saw annual cases drop from roughly 34,000 to under 400 by the early 1990s [191]. The United States saw over a 99% decrease in MuV cases—from over 150,000 annual cases in 1968 to fewer than 300 by 2003 [192].

Despite high vaccination coverage, a notable rebound in mumps has occurred in many countries since the mid-2000s. In Canada, outbreaks in 2007, 2010, and 2016–18 have totaled to over 7300 cases—predominantly in adolescents and young adults aged 15–29 [190]. In the United States, outbreaks initially reappeared from 2006 and onward with peaks in settings such as universities. Large epidemics in 2006 (~6600 cases), 2014 (~1150 cases), and 2016–17 (~2700 cases) have been documented [192,193]. Epidemiological and modeling studies have strongly suggested that waning immunity—averaging around 27 years post-vaccination—as the primary driver of these outbreaks, with about one-third of vaccinated individuals losing adequate protection by age 18 [194]. Genetic mismatches between circulating genotype G strains and the genotype A-based vaccine strain may play a minor role, but the age distribution of these newer cases supports waning immunity as a major cause [195].

These resurgent MuV outbreaks have tended to cluster in regions with high-density, close-contact environments like colleges, military barracks, and tight-knit communities. United States outbreaks in the Midwest and New York have largely affected university-aged individuals, often despite a documented high two-dose vaccination rate [187]. In Canada, genotype G has dominated nationwide with cases concentrated in those between the ages of 15–29 [190]. Europe has also seen a massive outbreak of 56,000 cases in England and Wales in 2004–05 among young adults [195]. The commonality across these outbreaks appears to be demographic with age-cohorts too old to have received two doses early in life, but too young to have acquired natural immunity, now exhibiting increased vulnerability due to possibly diminishing vaccine-induced protection.

In summary, the dramatic reduction in mumps cases following MMR introduction has been undercut in recent years by resurgence of MuV infections amongst young, vaccinated individuals. It is proposed that this resurgence is primarily driven by waning immunity rather than solely viral evolution—with outbreaks concentrated in close-contact settings across both North America and Europe.

### 5.2. Genotype Distribution and Evolution

MuV is classified into 12 genotypes (A through N, excluding E and M). This genotypic classification is based primarily on the hypervariable SH gene sequence, with further refinement using F and HN genetic data [196]. Historically, genotype A had dominated wild-type transmission before the 1990s—but in the 21st century this genotype has all but vanished from the wild and remains primarily present only in the Jeryl-Lynn vaccine strain formulation. Since roughly 2010, the circulating genotypes have been primarily C, D, G, H, I, F, J, K, and L. Genotype G has become the most prevalent genotype, with consistent detection in North America, Europe, Australasia, and parts of Africa. Genotypes F, H, and I are more prevalent in East Asia, and genotypes C, D, H, and J have appeared more frequently throughout the Western hemisphere [197,198].

While MuV has a single serotype, the virus undergoes subtle antigenic drift within key surface proteins HN and F—especially between vaccine-genotype A and circulating genotype G strains. Despite the virus’ relatively low mutation rate for an RNA virus, the SH gene shows upwards of a 21% nucleotide variation across genotypes, with HN and F accumulating mutations affecting neutralization epitopes [196,199]. Serological studies have reported significantly reduced cross-neutralization of genotype G strains by antibodies raised against the genotype A-based vaccine [176]. In China, the drift between wild-type genotype F and the vaccine strain has increased throughout the years, coinciding with lowered neutralizing immunity especially post-single-dose vaccination [199]. However, the extent to which these antigenic variances translate to real-world vaccination failure remains highly contested.

Genotypic shifts have also altered outbreak profiles in recent decades. For example, genotype F predominated in mainland China between 1995 and 2015, evolving through multiple transmission chains and showing a substitution rate for the virus around 2 × 10^−3^ substitutions per site per year. Meanwhile, in Europe and North America, most outbreaks since 2006 have been linked to genotype G, which frequently enters through international travel before causing clusters in college or communal settings [196,199]. Whole-genome analysis from New Zealand’s 2017 outbreak revealed genotype G strains with only 85% HN protein identity to the genotype A vaccine strain, including mutations in key epitope regions that potentially facilitated immunological escape [200]. Nonetheless, waning immunity over antigenic variation alone is thought to play a dominant role in this immune evasion, though evolving antigenic divergence likely compounds susceptibility in vaccinated cohorts and diminishes barriers to spread.

MuV continues to evolve via a diverse genotype landscape shaped by global and regional dynamics, with genotype G emerging as the predominant wild-type strain worldwide. While one serotype typically provides broad immunological defense, the genetic drift between genotypes A and G have subtly influenced immunity and reduced cross-neutralizing in some cases. However, waning immunity appears to be the primary driving factor in vaccinated-population outbreaks. Ongoing molecular surveillance to detect emerging genotypes to assess whether vaccine strain updates or booster strategies are warranted remains critical.

## 6. Vaccine Effectiveness and Challenges

### 6.1. Jeryl Lynn Vaccine Strain: Strengths and Limitations

#### 6.1.1. Basis of the Current MMR Vaccine: The Jeryl-Lynn Strain

The foundation of the current MuV vaccine within the MMR (measles-mumps-rubella) triplex formulation is the Jeryl-Lynn strain, which is a live attenuated MuV developed in the 1960s by Dr. Maurice Hilleman. Once approved in the US for widespread use in 1967, the Jeryl-Lynn strain became the backbone of MuV vaccination programs globally due to its favorable safety profile and high capacity to induce protective and effective immunity. Unlike earlier inactivated or experimental vaccines, this live attenuated version provided durable immunity after just one dose, making it a practical and effective public health tool [16]. Even more importantly, the Jeryl-Lynn strain belongs to genotype A, which has become a rarity in the wild since its vaccine rollout—highlighting a key feature of its limited genetic representation in the face of viral evolution.

#### 6.1.2. Breakthrough Infections and Outbreaks in Vaccinated Populations

While the widespread adoption of the two-dose MMR vaccine regimen has been instrumental historically in reducing the number of mumps cases for decades, outbreaks among fully vaccinated individuals have been increasingly reported over the past two decades. These breakthrough infections have highlighted key vulnerabilities in long-term mumps immunity and limitations of current vaccination strategies. Furthermore, these infections have occurred in populations with high vaccine coverage and intense close-contact interactions [201].

**Figure 6:**
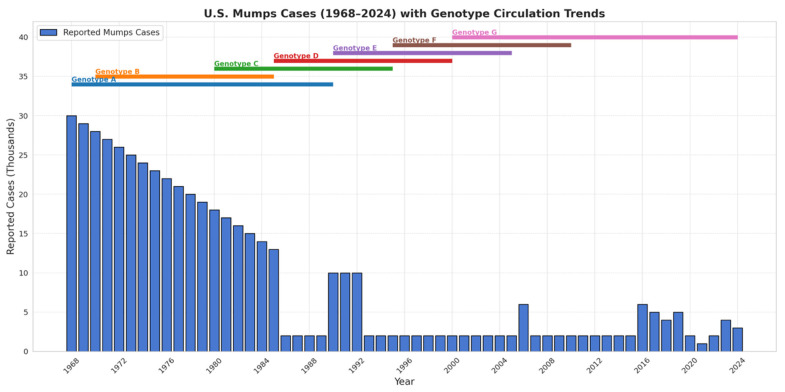
Timeline of US mumps cases from 1968–2024 with genotype circulating trends. The bars represent reported mumps cases in the thousands, while the colored lines at the top of the graph depict the approximate timeframes of dominant MuV genotypes (A–G) co-circulating clades in the US at the time based on historical surveillance. Notably, genotype G has dominated circulation since the early 2000s while the Jeryl-Lynn vaccine strain remains genotype A.

The most notable early example was arguably the 2006 outbreak in the US Midwest, with over 6000 cases reported—primarily among college-aged individuals who had received the recommended two MMR doses [202]. Subsequent outbreaks followed, such as those at the University of Iowa (2015–16) and Harvard University (2016). These two outbreaks followed a similar trend to their 2006 predecessor: the majority of cases occurred in individuals who had completed their vaccination schedules and were previously vaccinated with MMR [203,204]. In the 2016 Arkansas outbreak—one of the largest mumps outbreaks seen in the US in recent years—documented that over 93% of school-aged children who contracted mumps during that outbreak had already received two MMR doses [205]. This illustrated that high vaccine coverage with the current MMR vaccine strain does not necessarily prevent community-level transmission in high-density settings, at least when it comes to defending persons against currently circulating strains. This pattern is not unique to the US—similar outbreaks have been documented across several high-income countries with well-established MMR programs such as the UK, the Netherlands, and Canada. These repeated episodes have suggested that current vaccine-induced protection may be insufficient to prevent infection under conditions of high exposure or when immunological memory have waned. Figure 6 shows the epidemiological trends of documented mumps cases in the US over the last several decades alongside the prevalence of circulating MuV genotypes.

**Figure 7:**
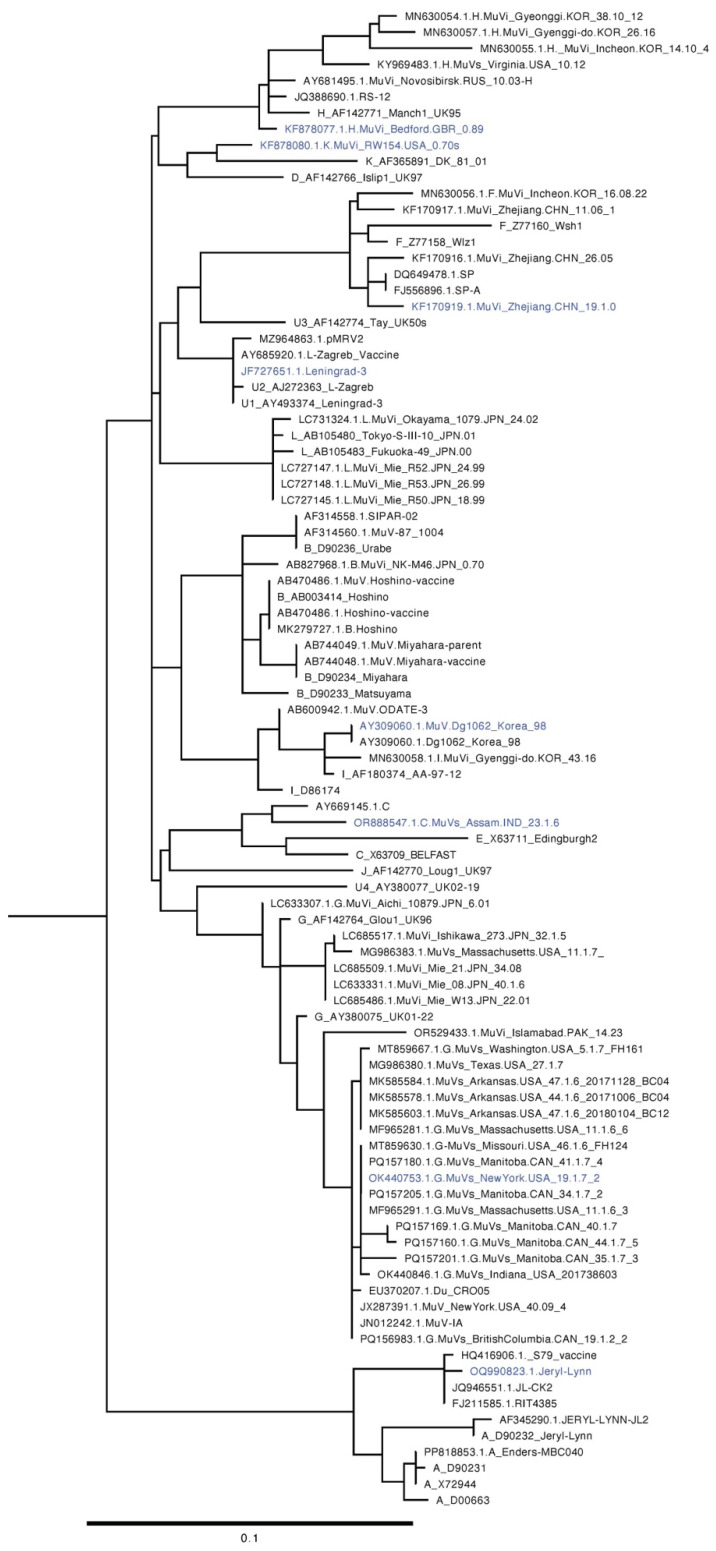
Phylogenetic tree of mumps virus genotypes A through G using short hydrophobic protein (SH) genetic analysis. Sequences in blue within the phylogeny tree were used as “reference sequences” when evaluating viral recombination events. Brian Foley, PhD of Los Alamos National Laboratory contributed this analysis and figure to this work [188,206].

Although mumps virus genotypes are labeled A through L, this alphanumeric nomenclature does not imply a chronological or evolutionary sequence. Phylogenetic analyses indicate that these genotypes diverged early and evolved largely in parallel, such that each genotype is approximately equidistant from early reference strains such as Jeryl-Lynn as seen in Figure 7. This branching pattern likely contributes to the continued cross-neutralization observed with the current vaccine strains, in contrast to the antigenic drift seen in influenza viruses [12,188,206]. Interestingly, breakthrough infections have been noted to be “less severe”, with vaccinated individuals experiencing milder symptoms and lower rates of complication compared to unvaccinated individuals. Despite this, they remain epidemiologically significant due to their role in sustaining transmission and seeding new outbreaks.

#### 6.1.3. Efficacy Against Historical and Contemporary MuV Strains

In the post-vaccine era after the introduction of the Jeryl-Lynn-based MMR vaccine, a dramatic drop in mumps incidence across many countries was observed. Population level studies and clinical trials initially estimate vaccine effectiveness between 78 and 88% after two doses [202]. However, though the vaccine was highly effective against earlier circulating wildtype strains, contemporary outbreaks in the current century have increasingly involved divergent genotypes, most notably genotype G with global dominance and prevalence. Numerous studies have exhibited that nAbs induced by current MuV vaccination protocols (utilizing the Jeryl-Lynn strain) exhibit reduced cross-neutralization capacity against circulating variants and budding genotypes [17]. While full immune escape is unlikely, it is generally accepted that increased antigenic mismatches can lower vaccine-induced protection effectiveness and could account for the resurgence of mumps cases seen even in highly vaccinated populations [207]. This has been increasingly evident in outbreak settings such as universities and camps, where not only close contact facilitates transmission of more virulent or antigenically distinct strains, but the younger adolescent populations appear to be an uncommon age group predominantly affected by this respiratory virus.

#### 6.1.4. Waning Immunity and the Booster Dose Debate

There is a growing body of evidence suggesting that Jeryl-Lynn-based MMR vaccine immunity waning over time. This waning immunity has been hypothesized to be a major contributor to the mumps outbreak resurgence in adolescents and young adults. Epidemiological modeling by Lewnard and Grad estimated that protection significantly declines approximately twenty-seven years after the second MMR dose, with a substantial portion of the vaccinated population becoming susceptible in early adulthood [208]. This waning immunity, coupled with genetic mismatch between the vaccine strain genotype A and currently circulating genotypes, has fueled a renewed debate over whether a third MMR dose (booster) should be recommended in routine or outbreak settings. Evidence from the 2016 US outbreaks supports the usage of a third dose, which was associated with a significant reduction in attack rates during university outbreaks [203,204]. The US Centers for Disease Control currently conditionally recommends a third dose in outbreak contexts, though routine booster usage remains under review [193]. Concurrently, there are some researchers that advocate for the development of new MuV vaccines, either as a third dose booster or new regimen entirely, that incorporate genotype-specific antigens or conserved epitope-based designs to improve long-term effectiveness. These developments underscore the importance of aligning vaccination strategies with evolving viral epidemiology.

### 6.2. Future Vaccine Strategies

#### 6.2.1. The Need and Design Considerations for Future MuV Vaccines

Current licensed MuV vaccines were developed decades ago using genotype A, yet the current globally predominant circulating MuV strains in the 21st century belong to genotype G and F [209]. Several experimental vaccine candidates derived from these current genotypes have exhibited higher neutralizing antibody titers in preclinical models compared to the traditional Jeryl-Lynn strain with genotype G- and genotype F-based vaccines outperforming genotype A vaccines in cross neutralization assays in mice [185,210]. A randomized trial in Chinese infants found that a genotype-F attenuated vaccine (SP strain) was both safe and immunogenic, offering noninferior responses compared to the Jeryl-Lynn strain standard [211,212]. These data underscore the public health rationale for development of genotype-matched vaccines to close the immunity gap being driven by antigenic mismatch.

While live attenuated vaccines, such as the Jeryl-Lynn strain, traditionally offer as close to a “real” infection to the immune system of the host as possible, there are safety considerations. Modern research exploring alternative platforms, such as reverse-genetic modified MuV strains using recombinant viruses lacking immunomodulatory V or SH proteins have been shown capable of eliciting robust neutralizing antibody and T cell responses in animal models [210]. DNA, subunit, and mRNA or self-amplifying RNA (saRNA) platforms are also being explored as methods to lower vaccine dosing and allow for broad, efficient antigen development on a timely production scale. Moreover, leveraging the MuV live-attenuated backbone for vector-based vaccines has shown promise—recombinant Jeryl-Lynn virus expressing a stabilized SARS-CoV-2 spike protein could generate potent systemic and mucosal immunity in both mice and hamsters [212]. Intranasal delivery in these systems also induced high levels of mucosal IgA and lung-resident T cells, which is an objectively attractive feature for respiratory virus protection that conventional subcutaneous vaccines may lack [213].

Beyond genotype-matching, next-generation strategies can also aim for universal or multivalent mumps vaccines. Heterologous prime-boost regimens using genotype F inactivated vaccines after priming with Jeryl-Lynn have shown enhanced cross-protection in mice with stronger cellular and humoral immunity than a homologous boost provided alone [185]. Furthermore, multivalent designs combing antigens from multiple genotypes within a single formulation could better span antigenic diversity to reduce breakthrough risk and stimulating more diverse and longer lasting immune responses [210,212].

It is also critical to consider the interplay of humoral and cellular immune responses, as they both contribute to protection against MuV but play distinct roles at different stages of infection. Neutralizing antibodies—primarily targeting the HN and F glycoproteins—are the strongest correlate of protection against reinfection and are essential for preventing viral entry and reducing early viral spread [17,20]. However, once infection is established, clearance of MuV appears to rely more heavily on cellular immunity. Studies in humans and animal models have shown that CD8^+^ T cells are critical for eliminating infected cells, while CD4^+^ T cells support both cytotoxic activity and the maturation of high-affinity antibody responses [17,20]. Notably, individuals with impaired T-cell function often experience prolonged or more severe MuV disease despite having detectable neutralizing antibodies, underscoring the importance of cellular immunity in resolving active infection. Together, these findings indicate that antibodies are central to protection, whereas T-cell responses dominate viral clearance—an immunologic distinction with implications for vaccine durability and the design of next-generation MuV vaccines.

In summary, future mumps vaccine strategies are likely to center on genotype-matched live or inactivated vaccines to improve coverage, testing next-generation platforms for rapid adaptability and enhanced systemic and mucosal immunity, and designing multivalent or universal vaccines capable of inducing robust protection across genotypes and broader, longer-lasting cross-immunity. Continued investment in vaccine antigen design, structural immunology, and advanced delivery platforms is key to achieving more effective and durable mumps control in the future.

#### 6.2.2. Current New-Age MuV Vaccine Candidates

One major advancement in new-age MuV vaccine development has been the development of a recombinant MuV vaccine candidate carrying the F and HN antigens from genotype G strains. One candidate built on the Jeryl-Lynn backbone called “JL-G” has been tested in rhesus macaques, and JL-G induced an 18-fold higher nAb titer against genotype G virus compared to the Jeryl-Lynn vaccine alone. JL-G induced titers also showed improved cross-neutralization across multiple genotypes (B, C, H, K, N) with no adverse reactions observed [212,214]. Similar genotype-matched vaccines from US and Chinese isolates in mice demonstrated significantly stronger neutralizing responses than Jeryl-Lynn against homologous genotype viruses [210]. Additionally, mice primed with two Jeryl-Lynn doses and then boosted with a genotype-G candidate showed marked increases in anti-G neutralizing titers, indicating a potential for heterologous boosting strategies [173]. Genotype G matched live-attenuated vaccine candidates against mumps shows promise in both diversity and ease of implementation into the current vaccine regimen.

Another live-attenuated MuV vaccine strategy utilizes modifications of the MuV polymerase mRNA cap methyltransferase, or MTase domain, to enhance safety and reduce neurovirulence. In one study, multiple recombinant S-79 MuV mutants with point mutations at the L protein’s catalytic sites showed significantly reduced replication and plaque size in vitro, with an absence of lung pathology normally seen in cotton rat and mouse models [215]. This candidate represents precision attenuation of polymerase function to improve vaccine safety while maintaining immunogenicity.

Beyond genotype matching, several innovative approaches are under investigation using the MuV platform as a live viral vector for multivalent or intranasal vaccines. A notable example is recombinant MuV called “rMuV-preS-6P”, a recombinant MuV expressing a stabilized SARS-CoV-2 prefusion spike protein. In both mice and golden Syrian hamsters, this candidate elicited strong systemic and mucosal IgA responses, robust T cell immunity, and complete protection against SARS-CoV-2 WA1 and Delta strain challenges—even in animals with preexisting MuV immunity [212,213]. These findings support the potential of utilizing MuV-based platforms for multivalent pediatric vaccines.

Overall, genotype G matched vaccines yield superior neutralization of circulating MuV strains and are strong candidates for replacing or supplementing the current Jeryl-Lynn based vaccine regimen. Polymerase mutated strains offer the option of enhanced safety profiles while preserving immunogenicity, making them suitable for more vulnerable populations in need of immune protection. MuV vector platforms of both intranasal and multivalent constructs demonstrate flexibility and potential for broad coverage to other respiratory pathogens or other mumps genotypes. Table 1 provides an overview of these current next generation platforms of MuV vaccine candidates. These developments highlight the budding research into options that could assist in overcoming current MMR limitations such as waning immunity and antigenic mismatch, helping to pave the wave for next-generation mumps immunization strategies.

## 7. Conclusions and Future Directions

Mumps resurgence in vaccinated populations over the past two decades has reinvigorated global attention on the virus’ pathogenesis, immunity, and possible control strategies. In this review, we have detailed the synthesized molecular, immunological, and historical and current epidemiological evidence to present a nuanced understanding of MuV dynamics. While the introduction of the Jeryl-Lynn component of the MMR vaccine initially led to a dramatic decline in mumps incidence, accumulating data highlights the limitations of the vaccine in the current landscape in context of waning immunity and viral genotype evolution. Genotype G strains now predominate globally and have been implicated in numerous outbreaks with young adults in close-contact settings among vaccinated individuals. These epidemiological shifts correlate with documented reduced neutralization by vaccine-induced antibodies, especially when the time since last MMR immunization exceeds a decade.

From a molecular and immunological perspective, advances in our understanding of MuV antigenic drift, innate immune evasion, and the longevity of humoral and cellular responses post-vaccination underscore the need for updated vaccine platforms. Recombinant live-attenuated candidates, genotype-matched constructs, and subunit or mRNA-based approaches show promising preclinical efficacy for offering avenues of more robust and durable protection.

The persistence of breakthrough infections with emergency of genotype-specific immune escape affirms the need for integrated MuV surveillance. Comprehensive research is also required to better define correlates of protection, understand mucosal versus systemic immunity, and assess the feasibility of booster dose strategies or even universal mumps vaccines.

In conclusion, the evolving landscape of MuV epidemiology demands a proactive response leveraging multidisciplinary insights. Sustained investment in basic and applied research spanning virology, vaccinology, immunology, and public health will be essential to controlling mumps in the decades that lie ahead.

## Figures and Tables

**Table 1:** Overview of next-generation MuV vaccine candidates with their platform designs, key findings, benefits, and limitations. JL = Jeryl-Lynn, G = genotype G, nAb = neutralizing antibody titer, GMT = geometric mean titer [212,213,214,215,216,217,218,219].

Vaccine Candidate	Platform Design	Key Findings & Immunogenicity	Benefits	Limitations	Citation
JL-G (Genotype G chimeric vaccine)	Live-attenuated Jeryl Lynn backbone expressing genotype-G F and HN proteins	In rhesus macaques, JL-G stimulated sera showed 18-fold higher neutralizing antibody GMTs against genotype G compared to the standard JL vaccine; broader cross-neutralization across genotypes B, C, G, H, K, and N; no adverse reactions observed	Better match to circulating genotype G strains, induces broader neutralization	Still live attenuated, needs safety testing in humans	[214,217]
rMuV-∆V, -∆SH, and -∆V∆SH (SH/V deletion mutants)	Live-attenuated recombinant MuV deleting accessory immunomodulatory proteins V and/or SH (genotype G isolates)	rMuV-∆V∆SH in mice was stable and immunogenic; induced neutralizing humoral responses comparable to or better than parental vaccine with reduced neurovirulence	Enhanced safety via attenuation, potential for strong immunogenicity	Preclinical only, needs further evaluation in primates and humans	[216]
Mtase-mutated rMuV-S79 variants	Mutations in mRNA cap Mtase or SAM-binding site of polymerase in S-79 strain (genotype F, China)	Recombinant rMuV-S79 mutants (e.g., A1814G, D1917A, E1990A) showed further attenuation in cotton rats while inducing robust neutralizing antibodies and full protection upon wild-type challenge	Increased safety via reduced replication; immunogenic and protective in small animals	Strain F-based, early-phase, human data lacking	[215]
Prefusion-stabilized glycoprotein immunogens (preF and preF-HN)	Recombinant subunit vaccine using genotype G pre-fusion F trimers with/without HN protein, stable at 37 °C	In mice, prime-boost with preF-HN generated high-titer nAbs against genotype A and G; potential for mRNA or protein-based formulation	Scalable subunit or mRNA format, stable and broad immunogenicity	Preclinical, unknown results in humans	[218]
Intranasal trivalent MMS (measles, mumps, SARS-CoV-2)	Live-attenuated MuV and MeV vectors expressing prefusion SARS-CoV-2 spike (preS-6P), delivered mucosally (intranasal)	In rodents, induced strong mucosal IgA and systemic immunity to measles, mumps, and SARS-CoV-2 variants; proof-of-concept for multivalent platforms	Multivalent protection; mucosal delivery may block transmission	Not peer-reviewed for human MuV immunity, early-stage	[212,213,219]

## Data Availability

No new data were created or analyzed in this study.
